# The ambiguous role of partially protected marine protected areas in Australia: Results from a systematic literature review

**DOI:** 10.1371/journal.pone.0307324

**Published:** 2025-01-07

**Authors:** Genevieve A. C. Phillips, Emily Ogier, Ian Dutton, Neville Barrett, Nils C. Krueck, Klaas Hartmann

**Affiliations:** 1 Institute for Marine and Antarctic Studies, The University of Tasmania, Australia; 2 Centre for Marine Socioecology, The University of Tasmania, Australia; MARE – Marine and Environmental Sciences Centre, PORTUGAL

## Abstract

Marine protected areas (MPAs) are an important tool in helping to protect biodiversity in the oceans. Recent ratification of the Kunming-Montreal Global Biodiversity Framework (GBF) has ensured that globally we are committed to effectively protecting 30% of the world’s oceans by 2030, in MPAs. In Australia there is considerable interest in the potential benefits that partially protected areas (PPAs) may provide. However, a consistent definition of a PPA is currently lacking, and urgently needed to conduct quantitative analyses of PPAs. We conducted a systematic literature review to understand the current knowledge surrounding PPAs and their potential benefits. We define a PPA, characterise PPA implementation in Australia, and present results for the outcomes of PPAs in terms of ecological, economic, and social indicators. Our review suggests that although 45% of Australia’s marine environment is within MPAs, 61% of MPAs provide only partial protection. The Northern Territory (100%), New South Wales (81%), and Queensland (79.8%) have the highest percentage of MPAs that are partially protected, compared to Tasmania which has the smallest percentage of partially protected MPAs (13.12%). Tasmania also has the smallest percentage cover of MPAs (6.49% state waters). Most PPA management plans did not contain quantifiable Key Performance Indicators (KPIs) to be able to effectively monitor the progress of these PPAs against the stated outcomes. We find the benefits of PPAs to be ambiguous: PPAs generally provide benefits when compared to ‘open’ ocean, however this is not a consistent result. There are no PPAs that provide greater overall benefits when compared to fully protected MPAs. Only one state (South Australia) and the Commonwealth (Australian Marine Parks) are collecting publicly available baseline data to facilitate quantitative monitoring of PPAs. Contrary to fisheries management, there were no plans of action if the declared MPAs and PPAs failed to meet their declared objectives and goals. Some PPAs within Australia appear to be incompatible with conservation priorities according to the recent “MPA Guide” classification framework. This study highlights the need for clearer management rationale and plans for PPAs in Australia, as these comprise the majority of MPAs in Australia’s Exclusive Economic Zone.

## Introduction

Marine biodiversity declines are a global concern. Protecting areas of the ocean from destructive and extractive activities in marine protected areas (MPAs) is an important tool to help address this problem. MPAs should aid in effective conservation as they are areas of the ocean that are managed with the primary goal of protecting ecosystems and conserving biodiversity, while allowing for the potential for sustainable use of resources [1]. MPAs are classified according to their level of protection from destructive or extractive practices to strict protection from all activities. MPAs where no activities are allowed (even entering the area may be banned) are often termed ‘no-take’, ‘no-entry’ or ‘fully protected’ MPAs. Under the International Union for the Conservation of Nature (IUCN) protected area management categories system (IUCN Cat.) these MPAs fall in categories I, II and III. In contrast, MPAs that do allow extraction of resources are often referred to as “partially protected areas” (IUCN Cat. IV, V, and VI [1]).

Fully protected (no-take, and no-entry) MPAs prohibit all extractive activities (fishing, mining, and oil and gas exploration or extraction) to primarily conserve marine ecosystems and biodiversity [2]. Fully protected MPAs may also block access to the area (e.g., pink zones within the Great Barrier Reef Marine Park [3]). Fully protected MPAs potentially provide the greatest biodiversity benefits for marine ecosystems [4, 5], however these areas prohibit access for activities that could derive direct economic benefits from a particular area, such as sustainable extraction through fishing or mining of resources; offshore windfarms [6]; non-extractive tourism activities; and the use of resources for social reasons or based on Indigenous uses (such as use of marine resources for traditional ceremonies). Fully protected MPAs allow the realisation of non-use social and economic benefits (hereafter referred to an indirect benefits) in addition to biodiversity benefits. These include socio-ecological benefits from the ecosystem being undisturbed (also known as existence value); economic benefits from increased fisheries productivity; and the potential economic and social benefits arising from protection of natural capital afforded by an MPA that future generations may have an interest in using in unanticipated ways or continuing to protect (also known as bequest and option values). These values are encapsulated within the total economic value framework [7] and flows of benefits from ecosystems which are assigned value are described in the ecosystem services assessment framework [8, 9]. Importantly, fully protected and no access MPAs also serve as natural reference areas for research needed to inform marine management, including both sustainable fisheries management as well as biodiversity conservation planning, and impacts of climate change on the marine environment [10].

The IUCN established a global goal to effectively protect at least 30% of the world’s biodiversity on land, in rivers and lakes, and in the ocean by 2030. This is also known as the “30 x 30” goal. The IUCN 30 x 30 conservation targets have recently been ratified in the adoption of the Kunming-Montreal Global Biodiversity Framework (GBF) at the fifteenth Conference of Parties (COP-15) in December 2022 [11]. There are three main objectives to the framework: 1) to protect and conserve biodiversity, ecosystems, and genetic resources; 2) to promote the sustainable use of biodiversity and ecosystem services; and 3) to ensure the equitable sharing of benefits derived from the use of genetic resources. Additionally, there are 23 “action-oriented” targets, two of which relate directly to marine protection. These two targets (Targets 2 and 3) state that”30% of degraded habitats must be under effective restoration, and 30% of the earth (land, water bodies, and marine environments separately) must be effectively conserved and managed by 2030, adding to the “30 x 30” goals. Australia has recently re-announced its commitment to the 30 x 30 IUCN goals by signing the GBF, by way of response to the release of the marine chapter of Australia’s State of the Environment report that outlines effective protection is required for Australia’s marine environment [12]. In practice, these commitments mean that 30% of Australia’s waters are aimed to be under effective conservation management by 2030: they will be protected from all forms of destructive extractive activities, with implemented management plans outlining the goals of protection, and methods to monitor the effectiveness of protection.

As of May 2023, 8.26% of the ocean is within the boundaries of either implemented (80.5%) or proposed MPAs (19.5%). However, only 2.9% of the ocean is within fully, or highly protected MPAs [13]. As we approach 2030, MPA coverage is increasing, even if the level of protection and management within MPAs worldwide varies widely, thereby introducing inconsistencies in the overall effectiveness to biodiversity conservation [2]. The placement and local size of MPAs is also crucial to their success. If most MPAs are placed in areas of low economic interest or low biodiversity function [14], the stated benefits to species and ecosystems under the GBF may not be achieved, even if the IUCN 30 x 30 goals are met on paper. Similarly, if MPAs are small relative to the movement distances of resident species they aim to protect from certain threats, specifically fishing, MPAs may not contribute effectively to the population recovery and biodiversity conservation goals under the GBF [15–18].

In Australia, as in the rest of the world, many new, and large, fully protected MPAs are situated in offshore areas that are of low economic interest and may not reflect areas of high biodiversity Bond and Jamieson 2022; Devillers et al. 2015) [19, 20]. This is because implementation and maintenance of fully protected MPAs in remote or difficult to access areas generally leads to fewer conflicts with other marine stakeholders [14]. Fully protected MPAs proposed within inshore, highly biodiverse areas can lead to difficult trade-offs between multiple user groups, and often become highly politicised debates. Therefore, worldwide, there is considerable interest in implementing MPAs that allow for multiple uses including some extraction of resources. A global synthesis (albeit in 2015) suggested that the majority (95%) of MPAs worldwide allow some form of extractive use [21]. These ‘partially protected areas’ (PPAs) are attractive to decision makers as they may reduce conflict by allowing users to sustainably remove marine resources from MPAs (e.g., by allowing fishing, both commercial, recreational, and traditional/indigenous), and provide opportunities to test and apply allocation/access policies to determine which forms of use best meet social, ecological and/or economic objectives. There is increasing interest in the use of PPAs in the marine environment [22]. Allowing fishing in an MPA (thereby creating a PPA), may reduce the likelihood of fisheries effort shift into smaller spatial areas, thereby potentially reducing the risk of localised depletions of fisheries species [23, 24]. If activities are restricted, the ecosystem should still be more protected than ‘open’ waters, if permitted activities are consistent with biodiversity values [25].

In this systematic literature review, we have three objectives. We start by firstly defining PPAs and providing a characterisation of PPA implementation across Australia. Second, we classify Australian PPAs under both IUCN guidelines and the more recent MPA Guide classification system [2]. Third, we assess the current understanding of the a) theoretical, and b) quantified benefits of PPAs within Australia through both peer-reviewed scientific literature, and relevant government publications. We then conclude with a discussion of our findings in the context of global MPA implementation strategies and present some recommendations and ideas for improving marine resource management both in Australia, and globally.

## Methods

### Literature search strategy

We used protocols for performing systematic literature reviews [26–31] to design the BOOLEAN search terms used in this study and published a systematic literature review protocol [32] that outlines our methods in detail. Briefly, two main literature searches were performed—the first, a search of peer-reviewed scientific literature was performed within the Web of Science Core Collection. The second, a search of Australian State and Territory Government legislation, was performed within the relevant databases (S1 Table). Search terms for legislative documents were based on those used by a previously published paper that was looking at the drivers of protected area development in Australia [33]. We modified these search terms to include the phrases ‘marine protected area’ and ‘partially protected area’ as relevant search terms. The search terms and operators used in both searches were chosen as to not include words that could bias the results such as “benefits” or “disadvantages”. We tested the search terms and refined the terms so that they produced literature relevant to our research questions (e.g. we included terms such as “marine protected area” so that the search results did not include research in terrestrial and freshwater systems). The final BOOLEAN search terms used, and the databases searched are provided in S1 Table.

Not all legislative instruments are publicly available in Australia. For example, there are departmental policy documents which may be used to inform or guide the management of marine resources, but these are not always published or publicly available. We elected not to use such documents within this paper so that this study could be repeated later by anyone, rather than just those that have access to a potentially biased, and hard to access selection of internal government documentation.

We also performed directed searches on the Departmental websites of all Australian jurisdictions (including the Commonwealth) for data on MPAs and their management to allow us to characterise the current state of PPA implementation in Australia (S1 Table). We utilised publicly available data published on these webpages, the IUCN webpages for definitions of MPAs, and The MPA Guide [2] and website associated with The Guide for additional information [34].

The search was conducted in English as the scope of the literature review was limited to Australia, and Australian territories, and, as outlined in the protocol [32] we limited the searches to the previous 11 years (2011–2022). This timeframe was selected to ensure that we were looking at the most recent studies and management ideas within this field of research. This timeframe is of particular interest to marine resource managers as many spatial management tools have been implemented in these years in response to the Aichi Target 11 (10% of the world’s oceans being protected by 2020). This timeframe also only includes MPAs that are defined under the current IUCN definitions (which were defined in 2008 [1]).

### Peer-reviewed literature—Article screening

**Fig 1:**
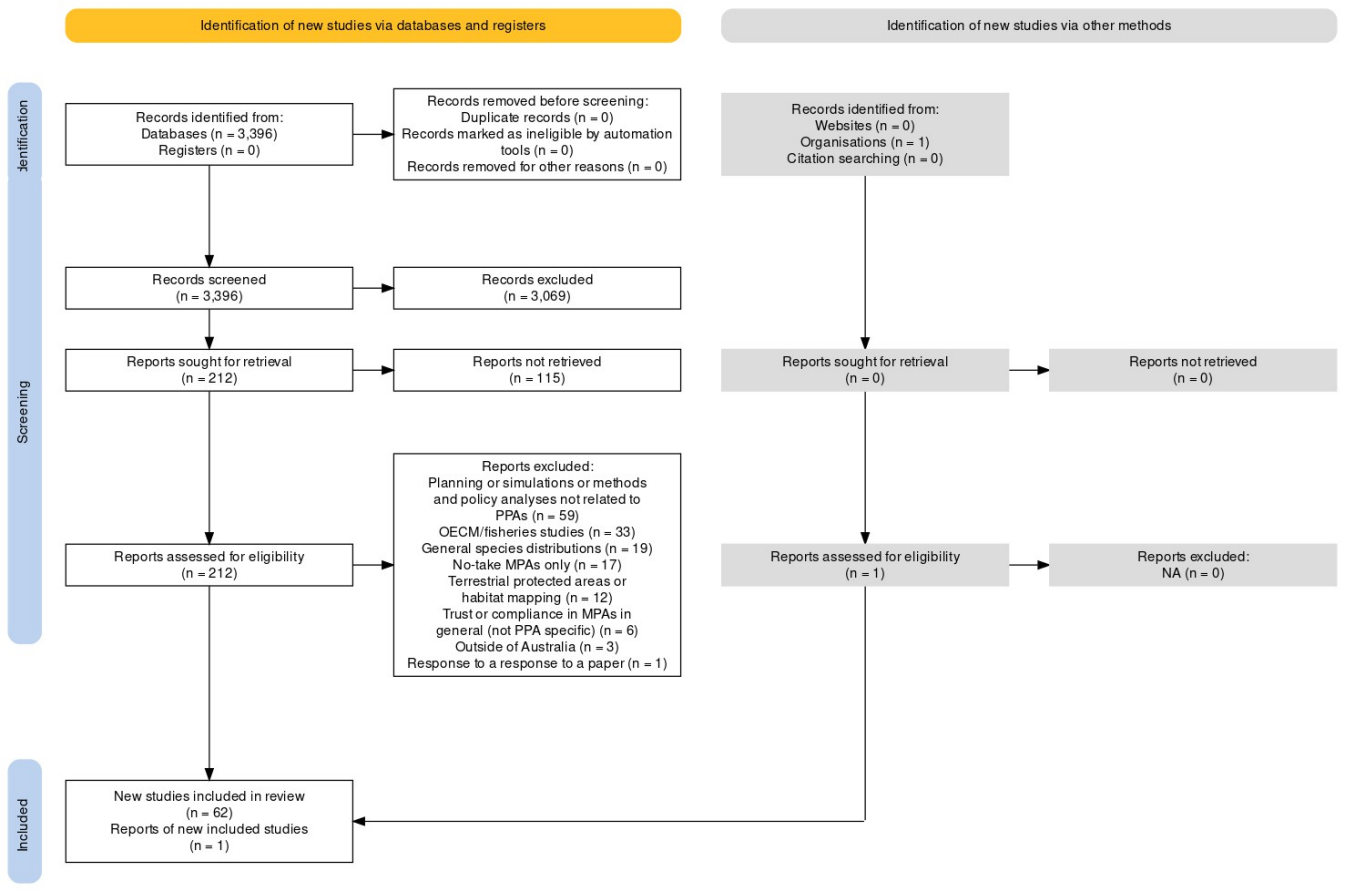
PRISMA diagram for the literature used within this review. The figure was generated using PRISMA2020 –an online Shiny app (https://estech.shinyapps.io/prisma_flowdiagram/) developed for systematic literature reviews that is compliant with PRISMA techniques [39]. Accessed May 2024.

The initial search of the literature produced 3396 articles. Articles were selected for in-depth reviewing by first reading every title in the returned articles and marking those articles for which the titles were considered to contain information relevant to the review. This was completed within the Web of Science interface, and articles that were marked were placed into a folder for further screening. Once all articles were screened by title, the next step was to read the titles and abstracts of the articles that had gone through the first screening process. Articles that were considered to contain relevant information for the literature review were marked and placed in a second folder within the Web of Science interface. These articles (212) were then read more extensively. Of the final 212 articles, 150 were rejected for final in-depth review as the content was not specifically about the costs and benefits of PPA implementation in Australia in the marine environment. For example, articles that were removed from the review at this point included articles only relating to no take MPAs (with no consideration of PPAs), articles concerned with terrestrial protected areas, and articles primarily concerning fisheries management areas. A PRISMA diagram (Fig 1) outlines the selection decision making process. One report investigating socio-cultural dimensions of MPA effectiveness in South Australia [35] was downloaded directly from a departmental website and is recorded in the PRISMA diagram in the right-hand side (articles identified using other methods). This report was downloaded as there was a published scientific paper that came up in the literature search and we wanted to have information from the primary document too. All relevant information for articles screened (title, authors, journal date published), the decisions made regarding an article (rejected, accepted), and the stage at which this decision was made (initial screening (titles); secondary screening (title and abstract); final screening and review (detailed reading of the paper)); and reasoning behind the decision to reject or accept the article in the review, were recorded in Excel spreadsheets.

We also trialled the use of the R package ‘*Revtools*’ in assisting screening the papers, as this was suggested as a quicker and more systematic process than manual sifting using the Web of Science Core Collection interface when our systematic protocol [32] was reviewed. However, we quickly found *Revtools* to provide a similar service to that of the WoS interface, with no added efficiencies, and so we retained our original method of using the Web of Science Core Collection interface to collect references, screen them, and save resulting queries, and articles.

### Metadata and data extracted

As described in the protocol [32], the following metadata and data were collected (titles, authors etc. were only collected once, the rest of the time the papers were referred to by their number assigned to them when downloading papers) and stored in Excel spreadsheets:

- Person screening the document.
- Article DOI
- Article type (e.g. peer-reviewed publication or legislative document)
- Article title
- Article authors
- State / Territory / area of Australia the study or legislation/policy is based.
- Protection classification of MPAs within article or legislation (based on The MPA Guide (27)
- Aspects of MPA studied if relevant (e.g. social consequences of implementing an MPA)
- Fisheries management and/or fisheries relevant regulations within MPA?
- Parks or conservation relevant regulations within MPA?
- Indigenous agreements or management regulations within MPA?
- Monitoring plan in place?
- What is being monitored? And how?
- Analyses of monitoring data?
- Outcome of monitoring / MPA implementation?
  - This relates to whether the MPA is delivering on the goals it has specified.
- Is MPA listed and visible on https://mpatlas.org/?
- Have there been any major changes to management of the PPA over time (if the PPA has been established for more than 10 years (i.e. prior to 2011))?
  - What are these changes?

### Literature search—Legislation

The Australian legislation database was screened using the BOOLEAN search terms outlined in S1 Table. Legislative documents were then screened to include any relating to marine protected areas and partially protected areas (if named directly). We also searched State and Commonwealth government department webpages to find publicly available policies or directions that may not be included in legislation, that related to MPAs in Australia. We used these searches to find information on management plans, and levels of protection associated with marine protected areas (to identify those that would be classified as PPAs), the intended benefits by declaration and management, and any available studies that may have been used to a) declare these protected areas, or b) monitor the effectiveness of PPAs in achieving intended benefits. We also screened websites for any information relating to the goals and predicted outcomes of MPAs in Australia, with a particular focus on PPAs.

### Categorisation of PPAs in Australia

All information on PPAs that was collected from the peer-reviewed literature and legislative documents (including policies and directions) was used to provide information on the current state of PPAs within Australia’s marine environment. We define a PPA within this paper, using information from The MPA Guide [2] and IUCN definitions for MPAs. Using our definition of a PPA, which can be applied globally, we identified Australia’s PPAs, and quantified their spatial extent, and the proportion of MPAs that are considered PPAs in Australia. These results are provided in Table 1 and the implications of these results are discussed in context with the primary and secondary research questions that this literature review attempts to answer. Additionally, we used data available from the Commonwealth Government [37] to characterise the extent (in km^2^) of MPA, PPA, and no-take MPA coverage across different jurisdictions, along with the percentage cover of jurisdictional water that these MPAs cover (Table 2).

**Table 1:** Data on marine protected area IUCN classification and coverage in Australia, as reported by state and territory governments and published by the Commonwealth of Australia [37]. IUCN categories I-III represent no-take MPAs (shaded blue), and categories IV-VI represent PPAs.

Jurisdiction Waters	IUCN Category	Area (km^2^)	Percent (%) area of jurisdiction waters in:			Percent (%) of jurisdiction MPAs that are PPAs.
			Marine Protected Area	Partially Protected Area	Fully Protected MPA	
Queensland	Ia	402.86	**51.6**	**41.9**	**12.0**	**79.8**
	Ib	-				
	II	14,243.59				
	III	-				
	IV	6,535.01				
	V	-				
	VI	44,555.20				
New South Wales	Ia		**39.63**	**32.1**	**7.5**	**81.0**
	Ib					
	II	662.43				
	III					
	IV	1,692.11				
	V	-				
	VI	1,133.94				
Victoria	Ia	-	**11.87**	**6.7**	**5.2**	**56.2**
	Ib	-				
	II	528				
	III	2				
	IV	-				
	V	-				
	VI	682				
South Australia	Ia	2,488	**49.45**	**39.2**	**10.3**	**75.9**
	Ib	-				
	II	3,670				
	III	6				
	IV	15,196				
	V	-				
	VI	8,324				
Western Australia	Ia	3,069.56	**41**	**30.1**	**10.8**	**73.8**
	Ib	-				
	II	8,765.56				
	III	630.59				
	IV	1,977.48				
	V	-				
	VI	33,084.53				
Northern Territory	Ia	-	**4.05**	**4.05**	**0.0**	**100.0**
	Ib	-				
	II	-				
	III	-				
	IV	-				
	V	-				
	VI	2,906.45				
Tasmania	Ia	947.66	**6.49**	**0.85**	**5.64**	**13.12**
	Ib	-				
	II	312.83				
	III	-				
	IV	11.37				
	V	30.20				
	VI	148.82				
Commonwealth	Ia	129,671.18	**45.08**	**27.2**	**17.8**	**60.6**
	Ib	-				
	II	1,413,789.53				
	III	-				
	IV	1,282,356.26				
	V	-				
	VI	1,095,922.70				
Total EEZ^^^		8,939,192	**45.08**	**27.2**	**17.8**	**60.6**

^^^Includes external territories of Norfolk Island, Christmas Island, Cocos (Keeling) Islands, and Heard Island and McDonald Islands. [2, 36]

**Table 2:** Categorisation of Partially Protected Areas (PPAs) in Australia alongside the MPA Guide Protection Level, and the reported IUCN Category for these zones. Extractive activities permitted are aggregated to each PPA name in the individual jurisdictions for conciseness. Industrial fishing is defined in The MPA Guide [2] as vessels > 12 m in length, which use towed or dragged gears. We have interpreted this as commercial trawling for benthic organisms such as scallops, prawns, or benthic fishes, as these fishing activities have the potential to have extensive ecological impacts if not well-managed, the vessels are generally > 12 m in length, and therefore the activities are incompatible with MPAs (as outlined in The MPA Guide [2].

Region	Partially Protected Area Zone Name	Extractive Activities Permitted	MPA Guide Protection Level	Reported IUCN Category
**Queensland**	Habitat Protection	No trawling, all other fishing.	Lightly protected	IV
	Conservation Park	Limited fishing and crabbing.	Lightly protected	IV
	General Use	Range of fishing, including trawling.	Incompatible (trawling)	VI
**New South Wales**	Aquatic Reserve**	Both recreational and commercial fishing permitted.	Lightly protected	IV
	Habitat Protection***	No trawling, most other fishing.	Minimally protected	IV
	Habitat Protection (Restricted)***	Additional restrictions to Habitat Protection Zones, including temporal closures to fishing.	Lightly protected	IV
	Special Purpose Zone	Highly restricted extractive activities. Aquaculture permitted.	Highly protected	VI
	General Use	Range of fishing. No setline / dropline, longlining, estuary mesh netting or purse seine netting. Trawling permitted.	Incompatible (trawling)	VI
**Victoria**	Marine and Coastal Park	Recreational and commercial fishing (no spearfishing).	Lightly protected	VI
**South Australia**	General managed use	All recreational fishing permitted	Highly protected (or lightly, depending on impact of recreational fishing)	VI
	Habitat Protection	All recreational fishing, no prawn trawling	Lightly protected	IV
	**Note: some shore-based recreational line fishing and or crab-raking is permitted in some Sanctuary Zones (*technically no-take zones*) and Restricted Access zones (*technically no-entry zones*) in South Australia. These sections of MPAs should be defined as PPAs.**			
**Western Australia**	Recreation	Recreational fishing ‘where appropriate’, subject to fisheries regulations; no commercial fishing.	Highly protected (or lightly, depending on impact of recreational fishing)	IV
	Special Purpose	Varies according to purpose of zone i.e., in Ningaloo Marine Park, shore-based recreational fishing is permitted in some Special Purpose Zones situated alongside Sanctuary (no-take) Zones. Other Special Purposes could be restrictions on lobster fishing, or ‘go-slow’ zones to protect sea lions, penguins, and dolphins.	Mixed—between highly and lightly protected	IV
	General Use	Sustainable commercial fishing, aquaculture, pearling, and petroleum exploration and production allowed. May have additional restrictions.	Incompatible (petroleum exploration & production)	VI
**Northern Territory**	Coburg (Garig Gunak Barlu) National Park	No nets or traps. Cast nets and hand spears permitted.	Highly protected	V
	Limmen Bight Marine Park	Some trawling allowed. All recreational and Indigenous harvest allowed. Commercial fishing permitted. Only seabed mining banned.	Incompatible (trawling)	VI
**Tasmania**	Marine Nature Reserves	Some areas within Marine Nature Reserves allow for restricted take of fish—these areas should be considered PPAs.	Highly protected	IV
	Marine Conservation Areas	Commercial and recreational fishing.	Lightly protected	VI
**Australian Marine Parks**	Recreational Use	Recreational fishing permitted.	Highly protected	IV
	Habitat Protection	No trawling, but most other commercial and recreational fishing.	Minimally protected^#^	IV
	Special Purpose	No trawling / fishing that disturbs the sea floor. Other fishing types and gears varies between Marine Parks. Mining (including exploration) permitted under class approval.	Incompatible (mining)	VI
	Special Purpose (Trawl)	Trawling permitted. Mining (including exploration) permitted under class approval.	Incompatible (trawling) (trawling + mining)	VI
	Multiple Use	Commercial and recreational fishing permitted, including trawling. Mining (including exploration) permitted under class approval.	Incompatible (trawling + mining)	VI
**Great Barrier Reef Marine Park**	Scientific Research (Orange)	Research (other than limited impact) and traditional fishing through permit.	Highly protected	Ia
	Buffer (Olive Green)	As above, but also trolling for pelagic species.	Highly protected	IV
	Conservation Park (Yellow)	No trawling / fishing that disturbs the sea floor. Several types of gear—both commercial and recreational.	Lightly protected	IV
	Habitat Protection (Dark Blue)	No trawling, but most other commercial and recreational fishing.	Minimally protected^#^	IV
	General Use (Light Blue)	Commercial and recreational fishing permitted, including trawling.	Incompatible (trawling)	VI

^#^ These zones meet the MPA Guide definition of ‘minimally protected’ as there are more than 10 gear types permitted.

* These areas are found within MPA zones that do not allow any extraction of resources (Sanctuary Zones or Marine Reserves), are regulated under both Parks Victoria and the Victorian Fishing Authority, and have not been declared as MPAs within the appropriate Act in Victoria, although they are treated as such within the CAPAD Database.

** New South Wales also has ‘Aquatic Reserves’ that are no-take zones (IUCN II). The ones in this table refer to the portions of ‘Aquatic Reserves’ that fall under IUCN IV category and allow various forms of recreational and commercial fishing. The fishing permitted depends on the specific reserve. For further information, see https://www.dpi.nsw.gov.au/fishing/marine-protected-areas/aquatic-reserves.

*** Habitat Protection Zones within New South Wales are found within Marine Parks (networks of MPAs) which are separate to ‘Aquatic Reserves’. There is extensive information regarding activities permitted within each zone in each Marine Park Network. For further information, see https://www.dpi.nsw.gov.au/fishing/marine-protected-areas/marine-parks.

### Mapping the current state of PPAs in Australia

We used geospatial data provided to the Commonwealth Government by State and Commonwealth departments in Australia, and our definition of a PPA to create a map of PPAs found within Australian waters. Data for marine protected area boundaries are publicly available and can be downloaded from the Collaborative Australian Protected Areas Database (CAPAD; https://fed.dcceew.gov.au/datasets/collaborative-australian-protected-areas-database-capad-2022-marine/). Australian jurisdictional boundaries are publicly available and sourced from Geosciences Australia (based on the Geocentric Datum of Australia 2020, GDA2020). Mapping was completed using spatial package *sf* [38, 39] within the R Programming Framework [38, 39]. All data are publicly available, and access to the data is via the Creative Commons Attribution (CC-BY) licence model:–CC By 4.0 International (https://creativecommons.org/licenses/by/4.0/).

## Results

In Australia, the understanding of the effectiveness and potential impact of partially protected areas in the marine environment (PPAs) is poor. The peer-reviewed literature we covered within this review provided varying results that were difficult to summarise due to a lack of congruency relating to indicators studied, and how these indicators were related to the PPA management plans.

The lack of understanding of the effectiveness of PPAs in Australia stems from three issues: 1) The lack of publicly-accessible management plans that have clear objectives and goals for PPAs (alongside no-take MPAs); 2) The lack of before-after-control-impact frameworks within analyses of the impact of PPAs; 3) The vast number and variety of PPAs in Australia, with no centralised or aggregated databases that collate both data collected on economic, social, cultural, and ecological metrics, and goals and objectives relating to the data. These issues are compounded by the limited resources available to monitor PPA performance; this is not a uniquely Australian problem [40, 41].

The results are structured to answer some of the secondary questions first, before summarising the results of the literature review. This structure provides clarity as to what a marine PPA is, before summarising the literature relating to marine PPAs in Australia.

### 1. Partially protected area (PPA) definition

Our study had several secondary questions that helped us answer the primary research question. Our first result was to provide a definition of a PPA that can be applied globally as there are no consistent definitions of PPAs in the literature. Our definition was based on information in The MPA Guide (REF MPA GUIDE) and the IUCN categories [1] and is used for the purposes of this study.

We define a partially protected area as:

*A partially protected area (hereafter PPA) in the marine space is a marine protected area (MPA), or spatial zone within an MPA Network, where extractive activities are permitted. PPAs may incorporate management rules and regulations relating to sustainable exploitation of resources within the MPA but are primarily regulated through conservation-based policy and legislation. PPAs are equivalent to IUCN MPA classifications IV, V, and VI.*

[1, 2, 22]

Within the IUCN classifications and The MPA Guide, there are references to ‘industrial’ fishing. Within The MPA Guide, the definition for ‘industrial’ fishing is “vessels > 12 m using towed or dragged gears”, and other incompatible activities are oil and gas exploration, mining, and fishing with dynamite or poison [2]. The IUCN definitions for a MPA allow for the ‘sustainable use of marine resources’ and do not include specific types of gear. Here we have used The MPA Guide definition when categorising activities that are incompatible with MPAs, and hence, PPAs.

### 2. Current state of PPA implementation within Australian waters

Within Australia, 45% of jurisdictional waters are declared to be within marine protected areas (MPAs) [37], either within State or Territory waters, or within the network of Commonwealth Marine Parks (waters outside State waters, but within Australia’s national waters, Table 2). This includes MPAs in Australia’s external territories such as the Cocos (Keeling) Islands, Christmas Island, and Heard and McDonald Islands. Using our definition of PPAs, most of Australia’s MPAs (61%) are PPAs, making up a total of 28% of Australian waters (compared to 18% of Australia’s waters in no-take MPAs) [37].

Using our definition of a PPA, all Australian jurisdictions contain PPAs (Table 1; [37]). In all jurisdictions, conservation-based agencies declare and manage park zonation, and fisheries agencies enforce fisheries regulations within the zones. The Northern Territory (100%), New South Wales (81%), and Queensland (79.8%) have the highest percentage of MPAs that are partially protected, while Tasmania has the lowest percentage of MPAs that are partially protected (13.12%)–and is also the state with the smallest percentage cover of MPAs (6.49% state waters, Table 1).

**Fig 2:**
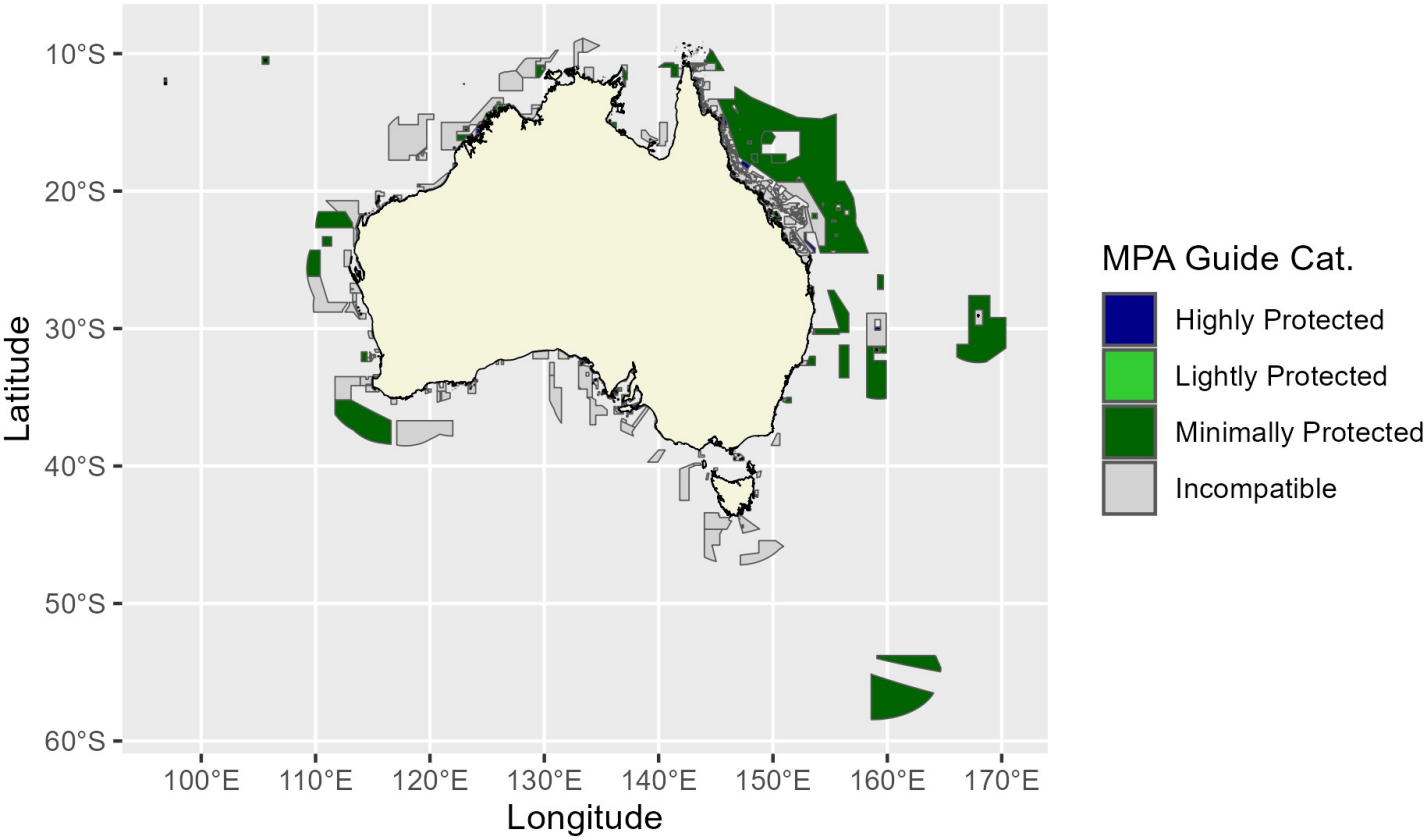
Australian marine protected areas (MPAs) categorised by this study using The MPA Guide categorisation system [2], based on data reported to the Australian Government. Dark blue areas are highly protected, light green areas are lightly protected, dark green areas are minimally protected, and grey areas are incompatible with the definitions of MPAs as categorised by The MPA Guide. All data relating to MPAs are sourced from the Collaborative Australian Protected Area Database (CAPAD) dataset for 2022 [36]. Australian jurisdictional boundaries are publicly available and sourced from Geosciences Australia (based on the Geocentric Datum of Australia 2020, GDA2020). All data are publicly available, and access to the data is via the Creative Commons Attribution (CC-BY) licence model:–CC By 4.0 International (https://creativecommons.org/licenses/by/4.0/).

We collated the data used to produce a map of PPA implementation in Australia (Fig 2, For individual state maps, see S1–S8 Figs). In Table 2 we outline the PPA zone names, the declared IUCN category in the Collaborative Australian Protected Areas Database (CAPAD) [37], and the relevant MPA Guide category. All categories of MPAs in The MPA Guide are found in Australia (Table 2). Of the 28 PPA zone categories we defined (separated by jurisdiction), 8 zone categories were highly protected, 9 lightly protected, 3 minimally protected, and 8 PPA zone categories were incompatible (Table 2).

Within the network of Australian Marine Parks (Commonwealth waters), all zones are considered PPAs, except “Pink zones” (Preservation Zones; IUCN Ia) found within the Great Barrier Reef Marine Park (GBRMP), and “Green Zones” (IUCN II) for which there are no issued permits for limited impact research or traditional uses (Table 2). We found all ‘General Use’ zones, or IUCN Category VI PPAs in Australia (except Victoria) were incompatible with the definitions of MPAs in The MPA Guide as they allowed some form of destructive activity, for example trawling, petroleum exploration and mining (Table 2). This equates to approximately 1,186,075.64 km^2,^ or 13.3% of Australia’s EEZ (using area of IUCN Category VI as declared in the CAPAD database, Table 1), and 29.5% of the area of MPAs in Australia.

### 3. The stated goals of PPAs in Australia (including fair access to resources, implementation priorities, and management changes)

Several of our secondary questions were related to understanding the stated goals of PPAs in Australia, how they have been implemented (e.g. primarily for conservation or fisheries); and whether management plans had well defined goals and objectives [32] Most of Australia’s MPAs (including PPAs) have published publicly available management plans, however there are many that are old, or have not been updated for several years (Table 3). For those PPAs where management plans have been published, only South Australia and New South Wales have management plans with quantifiable key performance indicators (KPIs). Quantifiable KPIs could also be included in plans as ‘STAR’-type goals (Specific, Timely, Action-oriented, and Realistic), or SMART goals—specific, measurable, achievable, relevant, and time-bound. Western Australian MPAs had some quantifiable KPIs, and Tasmania had KPIs only for Macquarie Island Marine Park. However, this management plan was last updated in 2006, and the KPIs within this plan only relate to biodiversity indicators [42].

**Table 3:** Australian marine protected area management units, and information on their associated management plans, including whether they contain measurable or quantifiable Key Performance Indicators (KPIs) for economic, social, biodiversity, and Indigenous or cultural goals; action plans if the goals or objectives are not met; and the relevant over-arching legislation or policy management tool for the marine protected area management unit.

Region of Australia	Responsible Department	Marine Protected Area Management Unit	Contains PPAs?	Management Plan with measurable Key Performance Indicators?	1) Stated Goals in Management Plan for MPA? 2) Action Plan if Goals Not Met?				How managed?
					Economic	Social	Biodiversity	Indigenous or Cultural	
**Queensland**	Queensland Parks and Wildlife Service as part of Department of Environment and Science	Great Barrier Reef Coast	Yes	Partial: Not for whole MPA, just marine areas of: 1) Fitzroy Island National Park 2) Green Island National Park 3) Swain Reefs National Park **No KPIs**	N/A	N/A	N/A	N/A	Marine Parks Act (2004)
		Great Sandy	Yes	No	N/A	N/A	N/A	N/A	
		Moreton Bay	Yes	Partial * *Oyster Management Plan and Moreton Island Management Plans available, but not a Management Plan for Moreton Bay Marine Park **No KPIs**	N/A	N/A	N/A	N/A	
**New South Wales**	Department of Regional NSW (NSW Department of Primary Industries) Department of Planning and Environment (Environment and Heritage; Planning) Transport for NSW	**NSW Marine Estate** Encompasses six marine parks: 1) Cape Byron Marine Park 2) Solitary Islands Marine Park 3) Lord Howe Island Marine Park 4) Port Stephens-Great Lakes Marine Park 5) Jervis Bay Marine Park 6) Batemans Marine Park; and 12 aquatic reserves, alongside 62 marine and estuarine habitats that are within National Parks and Nature Reserves.	Yes	Yes: Marine Integrated Monitoring Program and monitoring and evaluation framework **with KPIs for stated goals.** Framework developed in collaboration with all agencies, and independent Marine Estate Expert Knowledge Panel.	1) Yes 2) No				NSW Marine Estate (marine.nsw.gov.au) has Marine estate management strategy, an integrated monitoring program, planning, and associated marine-based legislation.
**Victoria**	Parks Victoria	**13 Large Marine National Parks** 1) Discovery Bay 2) Twelve Apostles 3) Point Addis 4) Port Phillip Heads 5) Yaringa National Park 6) French Island 7) Churchill Island 8) Bunurong 9) Wilsons Promontory 10) Corner Inlet 11) Ninety-Mile Beach 12) Point Hicks 13) Cape Howe; and **11 Marine Sanctuaries** 1) Merri 2) The Arches 3) Marengo Reefs 4) Eagle Rock 5) Point Danger 6) Barwon Bluff 7) Point Cooke 8) Jawbone 9) Ricketts 10) Mushroom Reef 11) Beware Reef **Six Marine Parks (MP), Marine and Coastal Parks (M&CP), and Marine Reserves (MR)** 1) Bunurong MP 2) Corner Inlet M&CP 3) Nooramunga M&CP 4) Wilsons Promontory MP 5) Wilsons Promontory MR 6) Shallow Inlet M&CP	No	Management Strategy (2003–2010) has expired. Management Plans are published, but have not been updated (e.g., Point Danger has a management plan dated 2005). The six partially protected areas lack management plans, goals, objectives, and systematic monitoring. **No quantifiable KPIs**	1) Yes 2) No	1) Yes, but in the Marine and Coastal Act (2018) 2) No	1) Yes 2) No	1) Yes 2) No	Marine and Coastal Act (2018)
**South Australia**	National Parks and Wildlife Service as part of Department for Environment and Water	**Network of 19 Marine Parks**: 1) Far West Coast 2) Nuyts Archipelago 3) West Coast Bays 4) Investigator 5) Thorny Passage 6) Neptune Island Group (Ron and Valerie Taylor) 7) Sir Joseph Banks Group 8) Franklin Harbour 9) Upper Spencer Gulf 10) Eastern Spencer Gulf 11) Southern Spencer Gulf 12) Gambier Islands Group 13) Western Kangaroo Island 14) Southern Kangaroo Island 15) Lower Yorke Peninsula 16) Upper Gulf St Vincent 17) Encounter 18) Upper South East 19) Lower South East	Yes	Yes: Management, Evaluation and Reporting (MER) Plan that details baseline data collection for ecological and socioeconomic data collection. Baseline reports for each of the 19 MPAs released in 2016 and a five-year strategic evaluation (2012–2017). **Quantifiable KPIs for all MPAs**	1) Yes 2) Partial—Strategic review outlines whether observed changes are influenced by the Management Plan for the Marine Park and provides information on whether trends will maintain, improve, or degrade the status of a particular indicator. No direct indication of what action should occur to ensure status is either maintained or improved.				Marine Parks Act (2007)
**Western Australia**	Parks and Wildlife Service as part of Department of Biodiversity, Conservation and Attractions	There are three different types of marine reserves: **1) Marine Parks** where sustainable extractive uses are permitted; 2) **Marine Management Areas**–where conservation, recreational, scientific, and commercial purposes are allowed; and 3) **Marine Nature Reserves** (only 1) where fishing is not permitted.	Yes	Most have Management Plans **with KPIs**. Some have expired e.g., Jurien Bay (2015). New marine management plans are joint with Aboriginal people (e.g., Lalang-gaddam Marine Park Joint Management Plan 2022)–legislated under amendments to the Conservation and Land Management Act (1984)	1) Yes 2) No	1) Yes 2) No	1) Yes 2) No	1) Some do, now—amendments in 2022 under the CALM Act (1984) have helped here. 2) No	Conservation and Land Management Act (1984)
**Northern Territory**	Department of Environment, Parks, and Water Security	Two Marine Parks—Limmen Marine Park and Coburg Peninsula	Yes	Yes—one expired. **No KPIs.**	1) Yes 2) No	1) Yes 2) No	1) Yes 2) No	1) Yes 2) No	Territory Parks and Wildlife Conservation Act (1976) Cobourg Peninsula Aboriginal Land, Sanctuary and Marine Park Act (1981) Fisheries Act (1988)
**Tasmania**	Parks and Wildlife as part of Department of Natural Resources and the Environment	21 Marine Reserves covering marine nature reserves and marine conservation areas. **Marine Nature Reserves (contain fully protected MPAs):** 1) Governor Island * 2) Kent Group 3) Maria Island 4) Ninepin Point * 5) Port Davey 6) Tinderbox * 7) Macquarie Island **Marine Conservation Areas (allow fishing):** 1) Blackman Rivulet 2) Central Channel 3) Cloudy Bay 4) Hippolyte Rocks 5) Huon Estuary 6) Monk Bay 7) Opossum Bay 8) Port Cygnet 9) River Derwent 10) Roberts Point 11) Simpsons Point 12) Sloping Island 13) South Arm 14) Waterfall-Fortescue	Yes	Management Plans for Macquarie Island, Maria Island and Port Davey Marine Reserves. Old (Maria Island Plan is 1998, and was supposed to be updated every 5 years, or earlier) ^#^ Port Davey also covered by Tasmanian Wilderness World Heritage Area Management Plan (2016) **KPIs only for Macquarie Island (only biodiversity related)**	1) Yes 2) No	1) Yes 2) No	1) Yes 2) No	1) Yes 2) No	Living Marine Resources Management Act (1995) National Parks and Reserves Management Act (2002) Nature Conservation Act (2002) Tasmanian Wilderness World Heritage Area Management Plan (2016)
**Commonwealth Waters: Australian Marine Parks**	Parks Australia	**Seven networks of MPAs**: 1) Coral Sea 2) Indian Ocean Territories 3) North 4) North-west 5) South-east 6) South-west 7) Temperate East	Yes	Yes: Management Plans and Implementation Plans either published or in progress for all MPAs. Desired outcomes stated, but **no quantifiable KPIs.**	1) Yes 2) No	1) Yes 2) No	1) Yes 2) No	1) Yes 2) No	Environment Protection and Biodiversity Conservation Act (1999)

* In Tasmania, these Marine Nature Reserves do not include PPAs as part of their zoning. The rest of the Marine Nature Reserves contain PPAs.

^#^ Maria Island Plan update is now in draft (2024) [45]

The webpages of government departments tasked with managing MPAs and PPAs in Australia all contained mission statements stating a commitment to protecting biodiversity without stating what biodiversity related to (e.g., species, or ecosystems). Additional commitments are stated for achieving social and economic goals for these MPA networks without a) defining what these goals are, or b) how they will achieve them, beyond using positive trajectory words such as ‘improve’ or ‘increase’. All webpages also have statements to include Indigenous or Aboriginal knowledge into management plans, or to work towards providing access to resources for Indigenous people, however there were no stated KPIs that would allow for documentation or monitoring of whether these goals have been achieved. This is in direct contrast to other forms of marine resource management, such as fisheries management, where there are well-established performance-based approaches to sustainable management, such as Harvest Strategies [43, 44]

Within MPA management plans in Australia there are statements relating to fair access to marine resources, without detailing how this will occur, how access will be monitored, and without defining criteria relating to success or failure. Only the Commonwealth MPAs (Parks Australia) have used implementation plans as well as management plans.

The stated goals of PPAs in Australia all cite conservation benefits as the primary objective of the area, with sustainable use as a secondary objective. This aligns well with the IUCN category definitions [1].

### 4. The potential benefits for PPAs in Australia, and are these being realised?

Results from the literature review were ambiguous as to whether PPAs provide overall benefits or not. The only consistent result in the scientific literature was that there were no studies suggesting PPAs were worse than ‘open’ areas in terms of biological, economic, or social variables. Open areas of the ocean in this case are areas that are not included within MPA boundaries; however, they are still managed under other legislation, including but not limited to, fisheries legislation. Most studies evaluated success or otherwise of MPAs (fully protected MPAs or PPAs) in relation to highly specific biological variables with no clear link to the management plans or stated management goals (biological, social and/or economic) of these areas. Most of these biological studies were quantitative studies of fish populations inside and outside partially protected areas (PPAs), thereby testing the direct biological benefits, and potential indirect economic and social benefits of PPAs (in terms of increased biomass of specific species). Few studies mentioned the impact of the age of MPAs on their results. No studies directly quantified economic benefits or otherwise of PPAs compared to fully protected MPAs or ‘open’ ocean. Six studies used survey data to understand how PPAs impacted social indicators and a further three studies contained recommendations relating to increased use of social indicators in PPA implementation and increasing stakeholder engagement when designing or integrating MPAs into existing legislative frameworks. The rest of this section discusses in detail the biological, social, and economic impacts of PPAs.

#### Biological impacts of PPAs

Biological variables were the most likely to be studied in the literature (56 out of 62 papers and one report reviewed), while 10 papers investigated social indicators. One paper [46] investigated both the social and ecological indicators within PPAs. The biological impacts of PPAs in the marine environment are highly variable, and the outcomes appear to depend on what is monitored, how it is monitored, and where the study takes place. There was no consistency in results between studies, except for studies relating to Australasian snapper (*Chrysophryus auratus*) where protection was positively related to abundance. Depending on the species studied, PPAs had neutrally positive effects (no difference to fully protected MPAs), neutrally negative effects (no difference to open areas), positive effects (PPAs provide benefits compared to fully protected MPAs, or benefits compared to ‘open’ areas), or negative effects (PPAs do not provide benefits compared to fully protected MPAs).

Four studies showed that fully protected MPAs lead to larger, and greater numbers of snapper than in PPAs and ‘open’ areas [47–50]. The response to reduced fishing pressure is thought to be quick (2–3 years; [48]), however the effect of PPAs themselves on snapper was mixed, with three studies showing no difference between PPAs and ‘open’ ocean [48, 49, 51], and one study showing PPAs benefited snapper over ‘open’ ocean [50].

In addition to there being no consistency in results between the studies in this literature review, there was little consistency in the methods used to test the benefits of protection in the marine environment. Population numbers (absolute values) or indices of abundance were commonly used to compare individual species or groups of fish species between fully protected MPAs, PPAs, and ‘open’ areas. However, species were chosen mainly on their commercial importance (e.g., [50–57]), or high socio-ecological value (‘charismatic’ species, e.g. [58–60]) and importantly, results were not consistent between studies. One study suggested their results were confounded by a recruitment event [55], whereas most did not take this into account when comparing data from different spatial zones.

Most studies did not contain baseline data or use counterfactual testing to identify whether the impacts seen were due to management changes, or simply spatial differences between sites. Before-after-control-impact analysis (BACI) is an imperfect, but potentially valuable tool to understand the impacts of management changes within the marine environment. This is especially true when considering managed populations [61] and studies investigating the effects of PPAs in Australia would have benefited from this approach.

One study investigated the changes to recreational catches of several fish species after the implementation of a PPAs that allowed recreational-only catch (recreation only fishery, ROF), and found increases in recreational catch and effort (approx. 2% increase in total recreational fishing effort) between six and eight years after the removal of commercial fishing for that species in the PPA [62]. Trends in length, length greater than Legal Minimum Length (LML), and the proportion of fish greater than LML were higher in the ROF compared to the non-ROF [62]. However, none of these trends were significant, and this was attributed to the short time frame of the study after management changes were implemented. *Plectropomus leopardus* (common coral trout) were found to be smaller [52, 63] and less bold [64] within PPAs compared to fully protected MPAs. Another study found some fish species had decreased abundance and length within PPAs that allowed trawling, compared to PPAs that had no trawling, however this trend was not universal even within the study, and the authors concluded that the benefits of PPAs are equivocal [56].

Commercially important fish species tended to benefit from some form of MPA (whether partially, or fully protected), and there was a trend towards increased biomass or mean length with an increase in MPA status (e.g. fish were largest, and/or most abundant in fully protected MPAs compared PPAs, compared to ‘open’ ocean), similar to results from global studies [5]. However, this trend was not consistent throughout all studies. Abundance of large fish in the ocean around Australia appears to have declined over time, regardless of whether found within an NTMR or PPA [65], although declines were less within PPAs (18%) compared to fished areas (36%), suggesting PPAs provide protection to large, commercially targeted fish [50, 65, 66], although in one paper this trend was not significant [67].

Unexploited species mainly showed ‘neutrally positive’ benefits in PPAs compared to fully protected MPAs (e.g. there were no significant differences in parameters measured between PPAs and fully protected MPAs), but also showed positive benefits in PPAs compared to fully protected MPAs if fish fauna diversity was used as a metric [68]. In one paper, a positive impact of PPA implementation was demonstrated for a population of critically endangered seahorses in PPAs compared to fully protected MPAs [69], which was attributed to the probability that seahorse predator abundance in the fully protected MPAs had increased since MPA implementation. One study [70] found that PPAs performed similarly to fully protected MPAs when relative numbers of endemic, near endemic, or protected species were used as an index for MPA “success”. Three studies found that PPAs may provide greater benefits than fully protected MPAs to highly mobile species such as sharks [58, 60], or predatory birds, particularly if the ‘open’ areas of ocean were well managed [59].

Biodiversity (using a new metric, zeta diversity) was found to be less stable within PPAs and ‘open’ areas compared to fully protected MPAs, with no greater improvements within PPAs compared to ‘open’ areas [71]. However, the result was no longer consistent (biodiversity was similar in PPAs, ‘open’ areas, and fully protected MPAs) when alpha and beta diversity (traditional metrics for measuring biodiversity) were used as biodiversity metrics as opposed to zeta diversity [71]. In fact, PPAs contained similar biodiversity to both fully protected MPAs and ‘open’ areas in three studies [49, 66, 71]. Similarly, although biomass and abundance of fish were found to be higher in fully protected MPAs than fished areas, (PPAs) in 12 studies [48–51, 54, 55, 63, 65, 72–75]), this was only true when looking at targeted fish (e.g. commercial or recreationally important species, although see [74]). The results were heterogenous across MPAs, and not consistent among species [51, 54, 66, 72, 76, 77]. For example, Lethrinids were lower in numbers in PPAs compared to fully protected MPAs, whereas *Epinephelus* spp. (Serranidae) increased in numbers in PPAs compared to fully protected MPAs [72].

Heterogeneity in results across years is not necessarily related to management type [51], suggesting other factors are influencing fish abundance in specific years, like recruitment pulses [55], similar to results from broader studies on the impacts of natural processes (cyclones, and cyclone frequency) on MPA effectiveness [78]. Surveys of fish abundance on PPAs and fully protected MPAs before, during, and after a ‘catastrophic flooding event’ in 2011, showed fished reefs (PPAs) did not recover as quickly as unfished reefs (fully protected MPAs) within Moreton Bay Marine Park [79].

In estuarine reserves, some species of fish increased in abundance in PPAs but not in fully protected MPAs, whereas others (estuary perchlet and blue catfish) were found in higher abundances in fully protected MPAs compared to PPAs [80]. Two studies [67, 81] found no evidence of differences in fish community composition or taxonomic diversity between fully protected MPAs and PPAs, even when using trait-based multi-metrics [67]. Two other studies found fauna diversity to be higher in PPAs compared to fully protected MPAs [68, 82].

There was no effect of protection (either PPA or NTMR) on non-fisheries target species, as expected [49, 73]. Two studies [48, 49] found there to be no differences between PPAs and reference or ‘open’ ocean at protecting Australasian snapper populations. Another paper studied a range of species using survey data, and reported that there were similar numbers of fish, invertebrates, and algae within PPAs as in the ‘open’ ocean [46]. The same study [46] suggested there were no apparent additional social benefits to PPAs either [46]. This study did not consider the social benefits of still being allowed to fish or access the area compared to being prohibited from entering the area (no entry, fully protected MPA), or accessing the area for recreational fishing, for example.

Two studies suggested that species caught as bycatch (not targeted, but still retained by fishers) gain protection in PPAs, showing an increased abundance and mean length compared to ‘open’ areas [53, 63]. However, the level of response to protection was found to be related to the level of fishing pressure prior to the MPA establishment [53]. Luderick, (*Girella tricuspidata*) were shown to be significantly larger and more abundant in PPAs (reserves within Jervis Bay Marine Park network, established in 1998, that allow commercial fishing), however, these results were not consistent across all reserves in the same network [76].

Where fully protected MPAs were found to increase fish assemblages (in terms of biomass and diversity), this was not homogenous across all fully protected MPAs in a network [51, 66, 72], with sometimes only one NTMR area within the network accounting for the majority of increased biomass and diversity [66].

Only three studies investigated fauna other than fish [83–85], and these studies disagreed on the benefits of PPAs compared to fully protected MPAs. One study [83] found species diversity (of octocorals) to be higher in PPAs compared to highly protected MPAs, whereas another study investigating the biomass of all major trophic groups found PPAs to contain significantly less biomass than fully protected MPAs [84]. The final study [85] compared macroalgal coverage on coral reefs between PPAs and fully protected MPAs and found there to be no difference in algal cover that could be attributed to MPA implementation. None of these studies compared data collected during the study to data prior to establishment of the different protected areas.

In this literature review we did not find strong evidence to link the degree of partial protection to targeted fish length distributions [86], or abundance [51]. This was surprising, given the considerable evidence that well enforced fisheries closures impact both length distributions and abundance of targeted fish species (e.g., [87]). One study, however, showed that PPAs allowing trawling had larger and more abundant fish than ‘general use’ PPAs [56], while another study showed no difference in sea snake abundance in trawled and non-trawled PPAs [88], a particularly interesting result, considering sea snakes are common bycatch in the trawl fishery in Queensland [89]. One study showed species had higher biomass inside sanctuary zones that allow shore-based fishing, compared to other PPAs that allow more types of fishing [86]. Again, these results were only for certain species of fish (smaller bycatch species), and no difference was found between the biomass of targeted species in sanctuary zones allowing shore-based fishing (technically no longer a sanctuary zone—see Table 2), and PPAs allowing more types of fishing [86], and no data were collected prior to implementation of the zoning arrangements. Additionally, PPAs were shown to increase sustainable yields of commercially targeted sex-changing fish [90], and have the potential to provide as many ecological benefits as fully protected MPAs when habitats are well connected within an MPA network [91].

When comparing a PPA (closed to spearfishing for 12.5 years) with ‘open’ ocean or ‘control’ areas, one harvested fish species (*Cheilodactylus fuscus*, red morwong) was in higher density in the PPA compared to the ‘control’ areas, whereas another species (*Acanthopagrus australis*, yellow-fin bream) had higher population densities in the PPA shallow waters (< = 3.5 m) but not in deeper waters within the PPA (4–12 m) [92]. However, these differences did not extend to densities of small or legal-sized fishes, highly mobile species, or lesser-targeted species [92]. These differences could be due to the accessibility of the fish to recreational spearfishers in shallow waters outside the PPA, and possibly highlight variability in the species targeted by spearfishers (e.g. the red morwong may be less attractive to spearfishers compared to the yellow-fin bream). These differences are important to characterise and understand prior to implementation of specific rules relating to resource extraction within PPAs or MPAs.

Interestingly, sometimes fish assemblages were more accurately predicted by other variables that were not considered in other studies. For example, one study, [93], found that the most significant predictor variable for fish assemblage was fur seal activity, and not sea surface temperature, wave action, or management type (e.g. MPA implementation). Fish assemblages were significantly different inside sanctuary zones, with 119% and 45% more fish, and 49% and 19% more species of fishes inside sanctuary zones compared to PPAs [93]. On average, 34% more fish were found on shallow reefs inside sanctuary zones compared to outside, however when fur seal activity was included in models, these differences were no longer significant [93] highlighting the complexities of attempting to model ecosystems. Similarly, another study found reef complexity to be a strong predictor of fish abundance, and this was a stronger relationship on fished vs. unfished reefs [94]. In this study, the ‘unfished’ reefs are not ‘unfished’, as they still allow trolling, and therefore should be considered PPAs.

Behavioural interactions between species, although markers of healthy ecosystems, something that MPA management plans aim for, were only studied in one paper. The paper investigated important cleaner interactions on PPAs and fully protected MPAs and found the time cleaner wrasse (*Labroides dimidiatus*) spent interacting with clients was less on PPA reefs than NTMR reefs [95], however these results were confounded with differences in species, and size of species cleaned on each reef type [95].

Few studies mentioned the importance of the length of establishment of the MPA that they were investigating, despite this being a key factor in MPA success [5]. However, those studies that did account for timeframes post-establishment, suggested that fish were quick (2–12.5 years) to respond to partial protection (e.g. fishing) [48, 62, 92].

#### Social impacts of PPAs

The scientific literature was equivocal regarding the social benefits or impacts of PPAs. As with the biological impacts, there was limited congruency with regards to methods of measuring social impact of PPAs, making it difficult to summarise results. There were nine studies that fulfilled the criteria to be included in this review, one of these studies investigated both social and environmental impacts of PPAs [46]. The paper found there to be no positive direct social benefits of PPAs compared to fully protected MPAs, as the benefits of these areas were unclear to the public, or there was a general overestimation of the protection that PPAs provided [46, 96]. However, they did find that PPAs provided more indirect social benefits than the ‘open’ ocean [46] The same study suggests that social benefits to MPAs are limited to indirect benefits, including increased understanding or awareness of an area; the attractiveness of the area; “human usage”; and those arising from the perception of the success (“better marine life”) and/or level of protection of the area. The study showed that fully protected areas had twice as many SCUBA divers, 3.5 times as many snorkelers, slightly more (0.2 times) boating activities, and “almost no people” fishing compared to open areas, with no differences seen between open areas and PPAs. While these statistics show that more people are found within fully protected MPAs, this does not suggest that PPAs provide no benefits—as there were similar numbers of people fishing in PPAs compared to ‘open’ areas, thereby providing additional spatial area for social benefits (compared to if the area was closed to fishing), and thus addressing a threat to community benefit (lack of access; [97]).

There were three studies that investigated the familiarity, or knowledge of the existence of PPAs and MPAs [96, 98–101] as opposed to the direct impacts of PPA implementation within Australia. The knowledge and familiarity of PPAs, the zoning, and permitted activities within PPAs, is linked to how well MPAs are supported [98]. Higher levels of support or knowledge of PPAs and their impacts was shown to have knock-on positive impacts relating to levels of support of fully protected MPAs [99, 102].

One paper suggested that PPAs provide indirect social benefits that enhance ecological conservation outcomes, as by having PPAs, people are more likely to be satisfied with their access to marine resources, or space, and therefore respect the sanctuary zones (fully protected MPAs; [102]). The study highlighted that people were found to have two conflicting ‘worldviews’: 1) marine areas need protection and therefore fully protected MPAs are the ‘ideal’ form of management; and 2) coasts are places of social interaction and community use, therefore traditional and cultural use of the resources should be maintained and prioritised. Within PPAs, non-extractive social benefits arising from access to these areas also include surfing, snorkelling, and diving [103]. To achieve ‘harmony’ between these two worldviews, the authors suggest that the most valued aspects of the marine environment, including the coastline, should be maintained, and protected, with consideration given for the definition of ‘protection’. The authors also suggest that PPAs allow communities access to the marine environment for social interaction and resource extraction (primarily fishing), and are therefore important in helping achieve equitable outcomes, if fully protected MPAs are still present to satisfy the first worldview [102].

One paper investigated the direct impact of PPAs on people’s social wellbeing and found that negative impacts of an MPA network (including PPA zones) were restricted to extractive users (mainly recreational fishers), with an increase in negative psychological factors, such as fear, stress, betrayal, and discrimination being reported most frequently by fishers [100].

#### Economic impacts of PPAs

Within the scientific literature for Australia, there were no studies investigating the direct or indirect economic impact of PPAs. Four studies have investigated socio-economic impacts of MPAs, and have comprised: (a) an assessment of the regional economic impact of one specific MPA [104], (b) the socio-economic impact of part of a MPA management planning process (e.g. Solitary Islands Management Plan [105]), (c) an investigation as part of advocacy for marine parks [106] and / or (d) a study as part of a long term process of understanding the human dimensions of protected area use [107]. Although the data are not exclusively from non-PPAs, it was not possible to distinguish what aspects were related to PPAs.

## Discussion

The results of our systematic literature review are equivocal with regards to the potential benefits of marine partially protected areas (PPAs) in Australia. In the scientific primary literature, there were few published studies that directly tested the impacts of partial protection, and most of these studies only tested the impact on ecological indicators. This was despite marine protected areas (MPAs), and those that are PPAs, being set up with the aim of providing economic and social benefits alongside ecological benefits. No peer-reviewed literature had studied the direct or indirect economic benefits of these areas, and only nine studies had incorporated social indicators in their assessment of PPAs. We found there were few published studies in the primary literature with rigorous evidence on the impact of PPAs (or fully protected MPAs), and there was a lack of trophic groups studied, other than those important for commercial or recreational fishing activities. This finding is comparable to the review by Fraser et al. [108], that investigated the impact of fully protected MPAs compared to PPAs within the Great Barrier Reef Marine Park (GBRMP). Additionally, the policy context of PPA establishment, and management, including extractive activities permitted (e.g., commercial, or recreational fishing) was not mentioned in any study, despite this being a considerable driver of MPA implementation in Australia.

Of critical importance is the lack of accounting for timeframes post-MPA establishment within the peer-reviewed studies in this review. Most studies did not acknowledge the impact of the time of establishment on their results, despite this being a key factor driving MPA success [5]. Of note is that those studies that did account for timeframes post-establishment, suggest species can be quick (2–12.5 years) to respond to partial protection, particularly if they are common fisheries species [48, 62, 92].

Despite Australia’s MPA management plans stating that MPAs will have benefits not just ecologically, but also socially and economically, there was only one peer reviewed study that had examined more than just one indicator of success [46] in PPAs. The study investigated the social and ecological effectiveness of PPAs and found that there were no ecological or social benefits. However, a limited set of social indicators were applied (increased attractiveness of PPAs for human usage relative to fully protected MPAs and increased positive perception of MPAs of all types) and did not use standardised approaches to measuring human usage levels. Our review highlights the need for improved identification of social and economic objectives (relevant to diverse stakeholders), matching KPIs, and robust assessment frameworks.

A recent report on the social and economic benchmark indicators of MPAs [109], identifies potential indicators for social and economic success for MPAs in Australia (based on the Commonwealth MPAs—the Australian Marine Parks), and these methods should be evaluated as to their effectiveness across PPAs within state and territory jurisdictional waters, alongside other methods for estimating economic benefits of MPAs [110]. Considering the extensive data collection from commercial and recreational fishers across Australia, that for the most part has been collected pre- and post-establishment of MPAs and PPAs, this is clearly an area where future research could provide some further insights into the potential social and economic benefits of PPAs in Australia. There are also emerging opportunities for assessment of the benefits of temporary fisheries and research closures (e.g. Elephant Rock and North Bay Rock Lobster Research Areas, Tasmania [111]).

In general, commercially-important species (most commonly, Australasian snapper–*Chrysophrys auratus*) appear to be the most likely to benefit from PPAs [48, 57, 72, 92], however within most studies, baseline data were lacking, making it hard to disentangle the effects of MPA regulations (and therefore the existence or not of a PPA), natural variability in population dynamics, fisheries management actions, and basic spatial differences between the NTMR and PPA that were being compared. No studies explicitly controlled for these effects in either their experimental design, or statistical analyses, but some studies acknowledged that population dynamics (e.g. a recent spawning event) may have influenced their results [55]. Whether snapper disproportionately benefit from PPAs compared to other commercially important (fisheries) species is difficult to evaluate without similar numbers of studies on other commercially important (fisheries) species, especially as there were some studies that suggested PPAs were no better than ‘open’ areas for snapper [49]. Additionally, some species responded positively to fully protected MPAs (with increased biomass), due to fisheries management actions, not just MPA zoning [108, 112].

### Management plans

Management plans with quantifiable KPIs would allow marine resource managers to understand a) when goals were met, not met, exceeded, or whether the management plan was failing; b) what data are required to assess the effectiveness of the PPA; and c) what management levers or actions could be used to change the trajectory of biodiversity, economic, social, or Indigenous values within a PPA. An example of a review of terrestrial management plans, and their included KPIs, and associated SMART goals (Specific, Measurable, Achievable, Relevant, Timebound) could be applied to marine management plans within Australia [113]. Our search for management plans for MPAs in Australia highlighted the varying maturity of marine management in different jurisdictions, probably at least partially attributable to shifts in priorities in funding for conservation planning and management (Table 3). Some jurisdictions have MPA management plans with KPIs that are clear and provide ways of monitoring progress against the objectives and goals of the management plan (e.g., Western Australia, South Australia, some Australian Marine Parks, Table 3). However, in all these plans, there are no action plans if the goals are not met—this appears to be a clear gap in effective management of MPAs, especially if compared to, for example, harvest strategies for commercially important fishery species where action plans must be documented and implemented when species are judged to be at various levels of virgin (unexploited) biomass [43]. In this regard there are also opportunities for better integration of fisheries data with protected area management data.

### Future of analysing potential benefits of PPAs

Although this systematic literature review did not find equivocal evidence for the impact of PPAs on ecosystems, social, or economic indicators, these areas may still provide a range of benefits. Part of the issue in a lack of results are that marine management zones have been loosely categorised into three broad categories of zoning in most studies. These are no-take marine reserves (fully protected MPAs), partially protected areas (PPAs), and areas outside MPAs (termed ‘open’ ocean). This coarse level of categorisation ignores the complexity of management arrangements within MPAs, whereas in PPAs there are multiple possibilities for extractive activities, all with potentially different impacts on social, economic, and ecological indicators. Additionally, the ocean surrounding MPAs (‘open’ ocean) in Australia is not without restrictions—all jurisdictional aquatic water is covered by legislation that permits or excludes various activities, including the extraction of marine resources.

To fully understand the impact of management arrangements (whether within MPAs or outside), more targeted and systematic data collection is required for pre-determined indicators for ecological, social (including cultural), and economic health (or success). Prior to collecting data, we need to understand more broadly the underlying mechanisms that may contribute to ecological, social, and economic change within MPAs, to allow for viable and robust hypothesis-driven research. Additionally, inclusion of Other Effective Conservation Measures (OECMs) in assessing the benefits of marine management strategies is imperative to a comprehensive understanding of partially protecting areas of the ocean in Australia. This is because there is evidence that OECMs have the potential to be as effective (and sometimes more effective than MPA management) at meeting conservation objectives [114, 115].

The study has highlighted that it is essentially very difficult to evaluate the effectiveness of PPAs by any set of biological, social, or economic metrics as a broad group, as individual PPAs have been set up with differing goals and objectives, and allow for a variety of uses, and levels of resource extraction. For example, a PPA within an MPA network may have been set up to protect a specific species, whereas another may have been set up to facilitate the harvest of another species. Future studies on the effectiveness of PPAs therefore should measure their effectiveness against the management plans and intended goals of the specific PPA, which would require a greater level of policy maturity than is present in most Australian jurisdictions at present. Understanding the impact of PPA declaration and implementation on fisheries restrictions inside MPAs and the alternative (whether fisheries management impacts the declaration of PPAs) would be interesting to understand. For example, two questions could be a) Are fisheries restrictions inside PPAs altered to reflect conservation outcomes (e.g. restricted catch and/or size limits compared to areas outside PPAs)?; and b) Does fisheries management become more precautionary after the declaration of PPAs in order to help achieve conservation outcomes? The answers to these questions would help understand the potential impacts of PPAs on overall marine resource management (and conversely the effects of PPAs on fisheries management outcomes). Several PPAs in Australia have been set up as ‘habitat protection zones’ with the primary goal of protecting sensitive benthic habitats from fishing practices where gear is dragged along the benthos, and/or cause irreversible destruction of the sea floor. More impactful management analyses would be to conduct experiments to compare the health of the benthos in these habitat protection zones to comparable habitat outside the PPA using BACI- based analyses.

An important new category of marine protection and stewardship instrument in Australia are Indigenous Protected Areas (IPA) within Sea Country [116]. These areas have evolved from specifically being centred around indigenous land tenure to include multiple tenures, and the marine space (Sea Country). These areas are indigenous-driven, collaborative, and provide models for a protected area governance framework that can support contemporary management of MPAs and the marine space in general. Currently there are limited data available on the impact(s) of IPA implementation in Sea Country around Australia [117], and they are not considered within the Nationally Representative Marine Protected Areas (NRMPA) within the CAPAD database [37], however this is something that should be considered in future implementations of this systematic review.

Since completing this systematic literature review, we have been advised by employees within various State and Commonwealth jurisdiction government departments that data do exist on specific parameters relating to ecological, social, and economic effectiveness of PPAs in Australia. Additionally, there are reports that address some of the issues within this review. However, the objectives of this systematic review included opportunities for replication in the future, including the search parameters and primary focus on peer-reviewed scientific literature. However, we will continue to work with State and Commonwealth agencies to aggregate reports and data platforms to make these available to the public, other researchers, and after direction from the project Steering Committee to the other jurisdiction’s agencies. This will facilitate collaboration between university researchers, managers, and between jurisdictions, which is imperative considering the dynamic nature of marine species and the evolving nature of marine resource governance at all levels (see Australia’s National Sustainable Ocean Plan—[118]).

We encourage other researchers to repeat our systematic review in the future, to allow for further time that may provide clarity on the impact of PPAs in Australia. This will require well designed BACI studies to account for the wide range of PPAs and the activities permitted within them.

## Conclusions

Despite the caveats and study limitations, this study highlights the need for clearer management rationale and more explicit plans for marine protected areas (MPAs) in Australia, and in-particular for partially protected areas (PPAs) as these comprise the majority of MPAs in Australia’s EEZ. With increasing competition for Australia’s ocean space and the diversity of management approaches that have evolved that seek to provide for diverse management objectives there is a critical need for the specific and relative contributions and effectiveness of MPAs and PPAs to be systematically evaluated.

Data and management plans for PPAs need to be available firstly for transparency of marine management, and secondly for researchers to use, to allow systematic evaluations of PPA effectiveness against the specific management objectives of a particular PPA. Assessments of effectiveness of PPAs should be completed within meaningful time scales, and there should be plans for assessments to occur at regular intervals, with transparent reporting against objectives, and action plans for what to do if PPAs are not meeting their objectives. Examples of this work beginning in Australia are the Science Plan under the Commonwealth Government, and the Management Evaluation, Reporting, and Improvement (MERI) framework for the Australian Marine Park network [119]. State of the Environment Reports, particularly with a focus on Australia’s Marine Estate [12] have also made some contributions in this regard but these should draw from a more systematic and comprehensive database.

## Supporting information

S1 Table Databases used in the systematic literature search, and the corresponding BOOLEAN search terms applied in the search, and the date the search was performed. (DOCX)

S1 Fig Australian Marine Parks: Partially protected areas (PPAs) categorised by this study using The MPA Guide categorisation system [2], based on data reported to the Australian Government. All data relating to MPAs data are sourced from the Collaborative Australian Protected Area Database (CAPAD) dataset for 2022 [37]. Australian jurisdictional boundaries are publicly available and sourced from Geosciences Australia (based on the Geocentric Datum of Australia 2020, GDA2020). Mapping was completed using spatial package *sf* [38,39] within the R Programming Framework [120]. All data are publicly available, and access to the data is via the Creative Commons Attribution (CC-BY) licence model:–CC By 4.0 International (https://creativecommons.org/licenses/by/4.0/).
(TIF)

S2 Fig New South Wales (NSW) PPAs categorised by this study using The MPA Guide categorisation system [2], based on data reported to the Australian Government. All data relating to MPAs data are sourced from the Collaborative Australian Protected Area Database (CAPAD) dataset for 2022 [37]. Australian jurisdictional boundaries are publicly available and sourced from Geosciences Australia (based on the Geocentric Datum of Australia 2020, GDA2020). Mapping was completed using spatial package *sf* [38,39] within the R Programming Framework [120]. All data are publicly available, and access to the data is via the Creative Commons Attribution (CC-BY) licence model:–CC By 4.0 International (https://creativecommons.org/licenses/by/4.0/).
(DOCX)

S3 Fig Northern Territory (NT) PPAs categorised by this study using The MPA Guide categorisation system [2], based on data reported to the Australian Government. All data relating to MPAs data are sourced from the Collaborative Australian Protected Area Database (CAPAD) dataset for 2022 [37]. Australian jurisdictional boundaries are publicly available and sourced from Geosciences Australia (based on the Geocentric Datum of Australia 2020, GDA2020). Mapping was completed using spatial package *sf* [38,39] within the R Programming Framework [120]. All data are publicly available, and access to the data is via the Creative Commons Attribution (CC-BY) licence model:–CC By 4.0 International (https://creativecommons.org/licenses/by/4.0/).
(TIF)

S4 Fig Western Australian (WA) PPAs categorised by this study using The MPA Guide categorisation system [2], based on data reported to the Australian Government. All data relating to MPAs data are sourced from the Collaborative Australian Protected Area Database (CAPAD) dataset for 2022 [37]. Australian jurisdictional boundaries are publicly available and sourced from Geosciences Australia (based on the Geocentric Datum of Australia 2020, GDA2020). Mapping was completed using spatial package *sf* [38,39] within the R Programming Framework [120]. All data are publicly available, and access to the data is via the Creative Commons Attribution (CC-BY) licence model:–CC By 4.0 International (https://creativecommons.org/licenses/by/4.0/).
(TIF)

S5 Fig South Australian (SA) PPAs categorised by this study using The MPA Guide categorisation system [2], based on data reported to the Australian Government. All data relating to MPAs data are sourced from the Collaborative Australian Protected Area Database (CAPAD) dataset for 2022 [37]. Australian jurisdictional boundaries are publicly available and sourced from Geosciences Australia (based on the Geocentric Datum of Australia 2020, GDA2020). Mapping was completed using spatial package *sf* [38,39] within the R Programming Framework [120]. All data are publicly available, and access to the data is via the Creative Commons Attribution (CC-BY) licence model:–CC By 4.0 International (https://creativecommons.org/licenses/by/4.0/).
(TIF)

S6 Fig Victorian (VIC) PPAs categorised by this study using The MPA Guide categorisation system [2], based on data reported to the Australian Government. All data relating to MPAs data are sourced from the Collaborative Australian Protected Area Database (CAPAD) dataset for 2022 [37]. Australian jurisdictional boundaries are publicly available and sourced from Geosciences Australia (based on the Geocentric Datum of Australia 2020, GDA2020). Mapping was completed using spatial package *sf* [38,39] within the R Programming Framework [120]. All data are publicly available, and access to the data is via the Creative Commons Attribution (CC-BY) licence model:–CC By 4.0 International (https://creativecommons.org/licenses/by/4.0/).
(TIF)

S7 Fig Tasmanian (TAS) PPAs categorised by this study using The MPA Guide categorisation system [2], based on data reported to the Australian Government. All data relating to MPAs data are sourced from the Collaborative Australian Protected Area Database (CAPAD) dataset for 2022 [37]. Australian jurisdictional boundaries are publicly available and sourced from Geosciences Australia (based on the Geocentric Datum of Australia 2020, GDA2020). Mapping was completed using spatial package *sf* [38,39] within the R Programming Framework [120]. All data are publicly available, and access to the data is via the Creative Commons Attribution (CC-BY) licence model:–CC By 4.0 International (https://creativecommons.org/licenses/by/4.0/).
(TIF)

S8 Fig Queensland (QLD) PPAs categorised by this study using The MPA Guide categorisation system [2], based on data reported to the Australian Government. All data relating to MPAs data are sourced from the Collaborative Australian Protected Area Database (CAPAD) dataset for 2022 [37]. Australian jurisdictional boundaries are publicly available and sourced from Geosciences Australia (based on the Geocentric Datum of Australia 2020, GDA2020). Mapping was completed using spatial package *sf* [38,39] within the R Programming Framework [120]. All data are publicly available, and access to the data is via the Creative Commons Attribution (CC-BY) licence model:–CC By 4.0 International (https://creativecommons.org/licenses/by/4.0/).
(TIF)

S1 Checklist PRISMA 2020 checklist. (PDF)
