# Supplementary material for: The ambiguous role of partially protected marine protected areas in Australia: Results from a systematic literature review
Source: PLoS One. 2025 Jan 7;20(1):e0307324. doi: 10.1371/journal.pone.0307324 (PMC11706464; doi:10.1371/journal.pone.0307324)

Supporting Information

S1 Table: Databases used in the systematic literature search, and the corresponding BOOLEAN search terms applied in the search, and the date the search was performed.

| **Database** | **Purpose** | **BOOLEAN search terms** | **Date performed** |
| --- | --- | --- | --- |
| Web of Science Core Collection | Primary peer-reviewed literature | **(TS=((marine protected areas) OR (partially protected marine protected areas) OR (partially protected MPA) OR (part* protec* marine protected area*) OR (partial protection) OR (partial protec* in MPA) OR (Sea Country) OR (Indigenous Protection Area) OR (Australi* Marine Protected Areas) OR (Australi* MPAs) OR (Australi* partially protected areas) OR (Australi* parti* protec* area*) OR (Australi* fisheries management) OR (Australi* sustainable fisheries management) OR (triple bottom line) R (soci* object*) OR (econ* object*) OR (biod* object*) OR (soci* goal*) OR (cons* object*) OR (cons* goal*) OR (econ* goal*) OR (biod* goal*) OR (cultural* object*) OR (cultural* goal*))) AND TS=((Aus*) OR (Austral*))** and **Article** or **Proceeding Paper** or **Review Article** or **Early Access** or **Correction** (Document Types) and **Environmental Sciences** or **Ecology** or **Marine Freshwater Biology** or **Environmental Studies** or **Fisheries** or **Economics** or **Water Resources** or **Oceanography** or **Biodiversity Conservation** or **Multidisciplinary Sciences** or **Geosciences Multidisciplinary** or **Law** or **Management** or **Green Sustainable Science Technology** or **Engineering Environmental** or **Geography** or **History** or **Sociology** or **Hospitality Leisure Sport Tourism** or **Geography Physical** or **Zoology** or **International Relations** or **Agronomy** or **Remote Sensing** or **Mathematics Applied** or **Evolutionary Biology** or **Parasitology** or **Social Issues** or **Biology** or **Behavioral Sciences** or **Ethics** or **Cultural Studies** or **Agricultural Economics Policy** or **Mathematics** or **Geology** or **Mathematical Computational Biology** or **Engineering Ocean** or **History Of Social Sciences** or **Engineering Marine** (Web of Science Categories) and **AUSTRALIA** (Countries/Regions) and **English** (Languages) and **3.275 Crop Protection** or **1.222 Epilepsy & Seizures** or **1.228 Virology - Tropical Diseases** or **1.7 Neuroscanning** or **3.87 Paper & Wood Materials Science** or **3.97 Plant Pathology** or **4.237 Safety & Maintenance** or **7.133 Geotechnical Engineering** or **1.137 Sleep Science & Circadian Systems** or **1.273 Health Literacy & Telemedicine** or **1.134 Trauma & Emergency Surgery** or **1.217 Parasitology - Malaria, Toxoplasmosis & Coccidiosis** or **1.26 Diabetes** or **4.17 Computer Vision & Graphics** or **6.238 Bibliometrics, Scientometrics & Research Integrity** or **6.321 Social Reform** or **6.69 Language & Linguistics** or **1.82 Gait & Posture** or **1.66 Hiv** or **10.240 Music** or **2.241 Membrane Science** or **3.198 Mycotoxins** or **7.139 Energy & Fuels** or **1.141 Hormone Therapy** or **1.150 Hearing Loss** or **1.248 Sexually Transmitted Infections** or **1.49 Dentistry & Oral Medicine** or **2.90 Water Treatment** or **5.131 Meteorological & Atmospheric Sciences** or **1.119 Breast Cancer Scanning** or **1.129 Back Pain** or **1.252 Smoking Cessation** or **1.36 Ophthalmology** or **1.55 Urology & Nephrology - General** or **1.68 Lipids** or **10.268 History & Philosophy Of Science** or **6.27 Political Science** or **6.263 Agricultural Policy** or **1.44 Nutrition & Dietetics** or **1.155 Medical Ethics** or **3.45 Soil Science** or **3.60 Herbicides, Pesticides & Ground Poisoning** or **8.8 Geochemistry, Geophysics & Geology** or **3.4 Crop Science** or **1.14 Nursing** or **1.156 Healthcare Policy** or **10.144 Modern History** or **4.224 Design & Manufacturing** or **6.11 Education & Educational Research** or **6.178 Gender & Sexuality Studies** or **3.51 Dairy & Animal Sciences** or **1.112 Palliative Care** or **1.172 Sports Science** or **1.21 Psychiatry** or **3.83 Bioengineering** or **4.183 Transportation** or **1.163 Parasitology - General** or **3.32 Entomology** or **3.91 Contamination & Phytoremediation** or **6.24 Psychiatry & Psychology** or **4.84 Supply Chain & Logistics** or **4.48 Knowledge Engineering & Representation** or **1.100 Substance Abuse** or **4.18 Power Systems & Electric Vehicles** or **1.72 Obstetrics & Gynecology** or **3.232 Veterinary Sciences** or **1.136 Autism & Development Disorders** or **1.246 Diarrheal Diseases** or **1.104 Virology - General** or **1.128 Fertility, Endometriosis & Hysterectomy** or **10.245 20th Century History** or **6.269 Political Philosophy** or **1.23 Antibiotics & Antimicrobials** or **6.185 Communication** or **7.229 Mineral & Metal Processing** or **10.290 Art** or **6.294 Operations Research & Management Science** or **9.92 Statistical Methods** or **3.220 Smell & Taste Science** or **3.275 Crop Protection** or **1.222 Epilepsy & Seizures** or **1.228 Virology - Tropical Diseases** or **1.7 Neuroscanning** or **3.87 Paper & Wood Materials Science** or **3.97 Plant Pathology** or **4.237 Safety & Maintenance** or **7.133 Geotechnical Engineering** or **1.137 Sleep Science & Circadian Systems** or **1.273 Health Literacy & Telemedicine** or **1.134 Trauma & Emergency Surgery** or **1.217 Parasitology - Malaria, Toxoplasmosis & Coccidiosis** or **1.26 Diabetes** or **4.17 Computer Vision & Graphics** or **6.238 Bibliometrics, Scientometrics & Research Integrity** or **6.321 Social Reform** or **6.69 Language & Linguistics** or **1.66 Hiv** or **1.82 Gait & Posture** or **10.240 Music** or **2.241 Membrane Science** or **3.198 Mycotoxins** or **7.139 Energy & Fuels** or **1.141 Hormone Therapy** or **1.150 Hearing Loss** or **1.248 Sexually Transmitted Infections** or **1.49 Dentistry & Oral Medicine** or **2.90 Water Treatment** or **5.131 Meteorological & Atmospheric Sciences** or **1.119 Breast Cancer Scanning** or **1.129 Back Pain** or **1.252 Smoking Cessation** or **1.36 Ophthalmology** or **1.55 Urology & Nephrology - General** or **1.68 Lipids** or **10.268 History & Philosophy Of Science** or **10.99 Literary Theory** or **2.244 Chemometrics** or **3.16 Phytochemicals** or **4.47 Software Engineering** or **6.314 Homelessness & Human Trafficking** or **7.192 Testing & Maintenance** or **7.300 Asphalt** or **8.212 Sensors & Tomography** or **1.111 Liver & Colon Cancer** or **1.125 Hepatitis** or **1.142 Urology** or **1.158 Dermatology - General** or **1.168 Vascular, Cardiac & Thoracic Surgery** or **1.179 Oncology** or **1.181 Molecular Toxicology** or **1.189 Genome Studies** or **1.194 Tuberculosis & Leprosy** or **1.199 Lung Cancer** or **1.231 Vitamin Metabolism** or **1.42 Bacteriology** or **1.52 Neurodegenerative Diseases** or **1.54 Molecular & Cell Biology - Genetics** or **1.65 Allergy** or **1.80 Bone Diseases** or **4.13 Telecommunications** or **4.322 Remote Research & Education** or **5.191 Space Sciences** or **6.277 Asian Studies** or **7.70 Thermodynamics** or **1.117 Pharmacology & Toxicology** or **1.120 Inflammatory Bowel Diseases & Infections** or **1.147 Prostate Cancer** or **1.154 Assisted Ventilation** or **1.157 Organ Donation & Transplantation** or **1.161 Virology - Identification & Sequencing** or **1.184 Physiology & Metals** or **1.186 Chromosome Disorders** or **1.218 Autonomic Regulation** or **1.247 Migraines & Headaches** or **1.304 Complementary & Alternative Medicine** or **1.34 Orthopedics** or **1.37 Cardiology - General** or **1.5 Neuroscience** or **1.94 Cardiac Arrhythmia** or **10.126 Philosophy** or **3.171 Photoproductivity** or **3.267 Virology - Plant** or **3.85 Food Science & Technology** or **4.187 Security Systems** or **4.284 Human Computer Interaction** or **4.29 Automation & Control Systems** or **5.20 Astronomy & Astrophysics** or **5.98 Geometrical Optics** or **6.256 Religion** or **7.121 Concrete Science** or **8.242 Nuclear Geology** or **9.50 Applied Statistics & Probability** or **1.105 Strokes** or **1.106 Rheumatology** or **1.108 Molecular & Cell Biology - Cancer & Development** or **1.148 Medical Mycology** or **1.164 Endocrinology & Metabolism** or **1.173 Cosmetic Surgery** or **1.196 Micro & Long Noncoding Rna** or **1.197 Molecular & Cell Biology - Mitochondria** or **1.203 Neuromuscular Disorders** or **1.235 Throat & Voice Disorders** or **1.25 Molecular & Cell Biology - Cancer, Autophagy & Apoptosis** or **1.258 Zoonotic Diseases** or **1.265 Dermatology - Skin Allergies** or **1.266 Wounds & Ulcers** or **1.285 Cystic Fibrosis** or **1.297 Asbestos & Mesothelioma** or **1.43 Anesthesiology** or **1.6 Immunology** or **1.75 Blood Clotting** or **2.296 Textile Chemistry** or **3.180 Microbial Biotechnology** or **4.116 Robotics** or **4.174 Digital Signal Processing** or **4.182 Data Structures, Algorithms & Complexity** or **4.289 Biophotonics & Electromagnetic Field Safety** or **4.46 Distributed & Real Time Computing** or **5.318 Physics Education** or **6.288 Information & Library Science** or **6.317 Risk Assessment** or **7.226 Electrical - Sensors & Monitoring** or **7.227 Manufacturing** or **7.260 Nuclear Engineering** or **7.63 Mechanics** or **9.143 Dynamical Systems & Time Dependence** or **9.162 Numerical Methods** (Exclude – Citation Topics Meso) and **Book Chapters** or **Editorial Material** (Exclude – Document Types) and **AUSTRALIA** (Countries/Regions) and **2022** or **2021** or **2020** or **2019** or **2018** or **2017** or **2016** or **2015** or **2014** or **2013** or **2012** or **2011** (Publication Years) | 3^rd^ March 2023 |
| Departmental Websites | Marine Protected Area Management Plans | Tasmania: Department of Natural Resources and the Environment - <https://parks.tas.gov.au/>  Victoria: Parks Victoria – <https://parks.vic.gov.au/>  South Australia: National Parks and Wildlife Service, South Australia - <https://marineparks.sa.gov.au/> and <https://data.sa.gov.au/>  Western Australia: Department of Biodiversity, Conservation and Attractions – <https://dbca.wa.gov.au>  Northern Territory: Northern Territory Government – <https://nt.gov.au>  Queensland: Department of Environment and Science – <https://des.qld.gov.au> and Great Barrier Reef Marine Park Authority <https://www2.gbrmpa.gov.au>  New South Wales: NSW Government – NSW Marine Estate - <https://www.marine.nsw.gov.au/>  Commonwealth Government: Parks Australia - <https://parksaustralia.gov.au/marine/> | March to June 2023 |

# S1-8 Figures:

Australian partially protected areas (PPAs) categorised by this study using The MPA Guide categorisation system [2], based on data reported to the Australian Government. All data relating to MPAs data are sourced from the Collaborative Australian Protected Area Database (CAPAD) dataset for 2022 [3]. Australian jurisdictional boundaries are publicly available and sourced from Geosciences Australia (based on the Geocentric Datum of Australia 2020, GDA2020). Mapping was completed using spatial package *sf* [4,5]within the R Programming Framework [6]. All data are publicly available, and access to the data is via the Creative Commons Attribution (CC-BY) licence model: – CC By 4.0 International (https://creativecommons.org/licenses/by/4.0/).

S1 Figure: Australian Marine Parks – PPAs categorised according to The MPA Guide

S2 Figure: New South Wales (NSW) PPAs categorised according to The MPA Guide.

S3 Figure: Northern Territory (NT) PPAs categorised according to The MPA Guide.

\

S4 Figure: Western Australia (WA) PPAs categorised according to The MPA Guide

S5 Figure: South Australia (SA) PPAs categorised according to The MPA Guide.

S6 Figure: Victoria (VIC) PPAs categorised according to The MPA Guide

S7 Figure: Tasmania (TAS) PPAs categorised according to The MPA Guide

S8 Figure: Queensland (QLD) PPAs categorised according to The MPA Guide


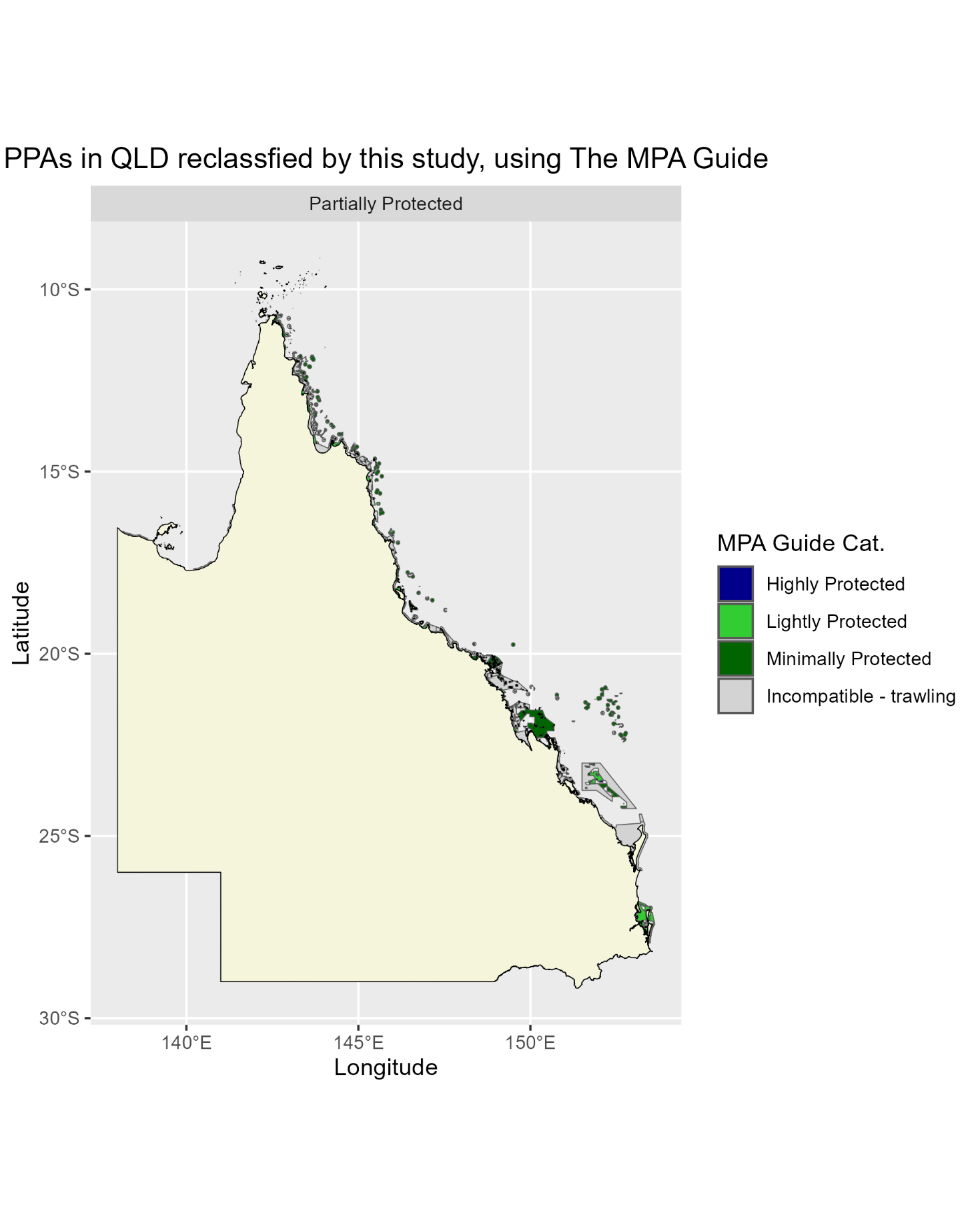

Supplement: S1 Table — (DOCX) [file pone.0307324.s001.docx]
