# Supplementary figures and images for: The ambiguous role of partially protected marine protected areas in Australia: Results from a systematic literature review
Source: PLoS One. 2025 Jan 7;20(1):e0307324. doi: 10.1371/journal.pone.0307324 (PMC11706464; doi:10.1371/journal.pone.0307324)

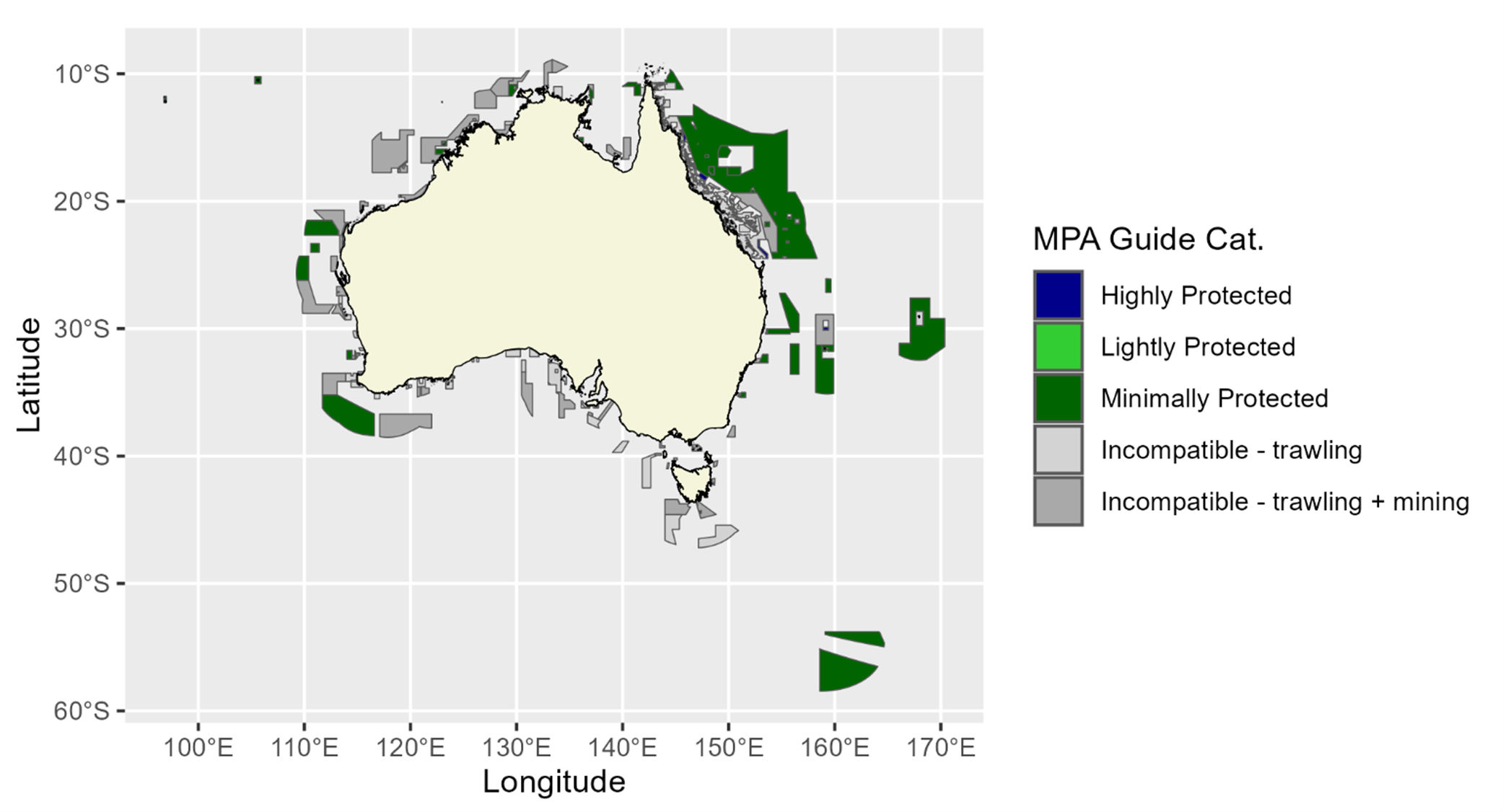

Supplement: S1 Fig — All data relating to MPAs data are sourced from the Collaborative Australian Protected Area Database (CAPAD) dataset for 2022 [37]. Australian jurisdictional boundaries are publicly available and sourced from Geosciences Australia (based on the Geocentric Datum of Australia 2020, GDA2020). Mapping was completed using spatial package sf [38,39] within the R Programming Framework [120]. All data are publicly available, and access to the data is via the Creative Commons Attribution (CC-BY) licence model:–CC By 4.0 International (https://creativecommons.org/licenses/by/4.0/). (TIF) [file pone.0307324.s002.tif]

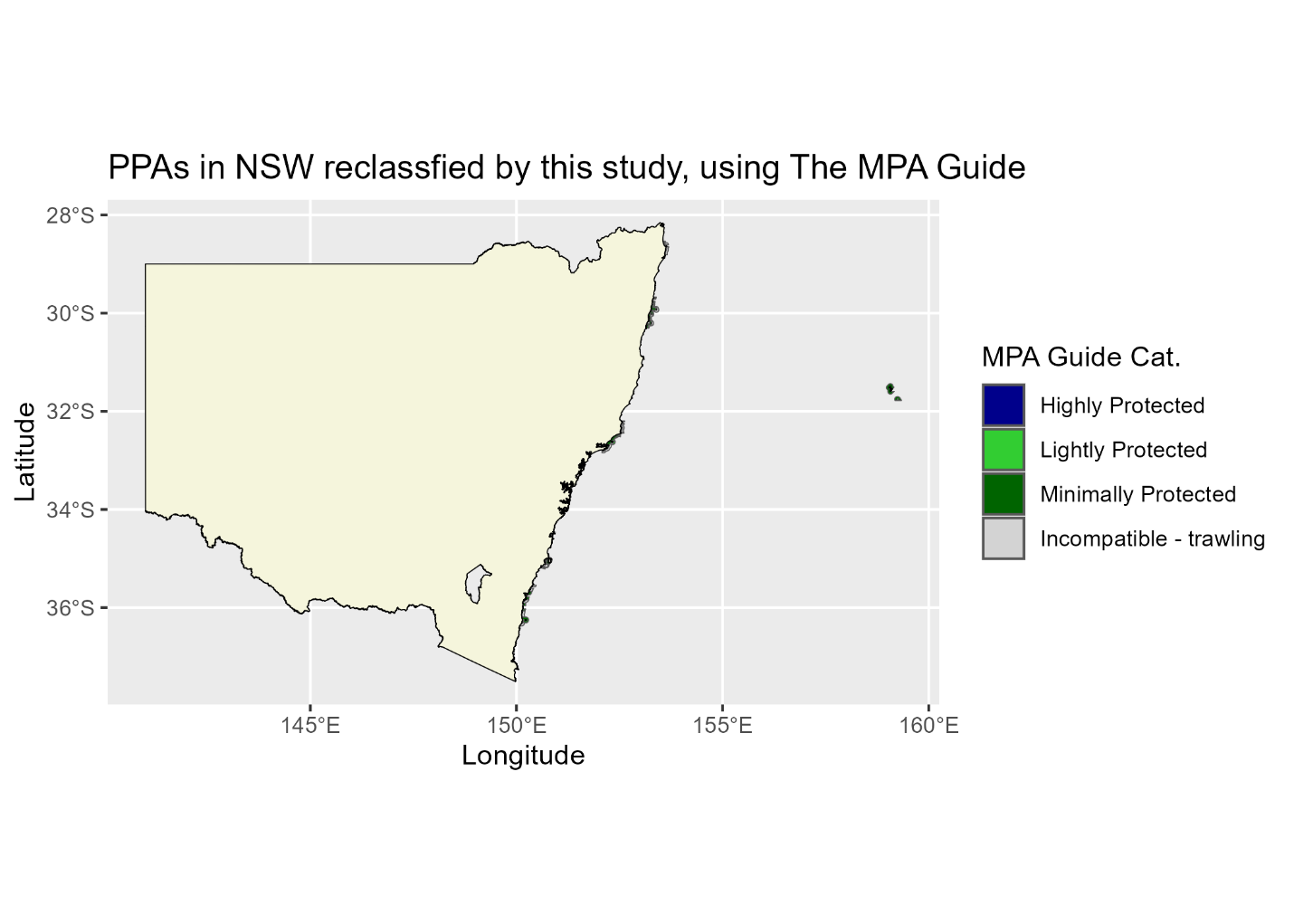

Supplement: S2 Fig — All data relating to MPAs data are sourced from the Collaborative Australian Protected Area Database (CAPAD) dataset for 2022 [37]. Australian jurisdictional boundaries are publicly available and sourced from Geosciences Australia (based on the Geocentric Datum of Australia 2020, GDA2020). Mapping was completed using spatial package sf [38,39] within the R Programming Framework [120]. All data are publicly available, and access to the data is via the Creative Commons Attribution (CC-BY) licence model:–CC By 4.0 International (https://creativecommons.org/licenses/by/4.0/). (DOCX) [file pone.0307324.s003.docx]

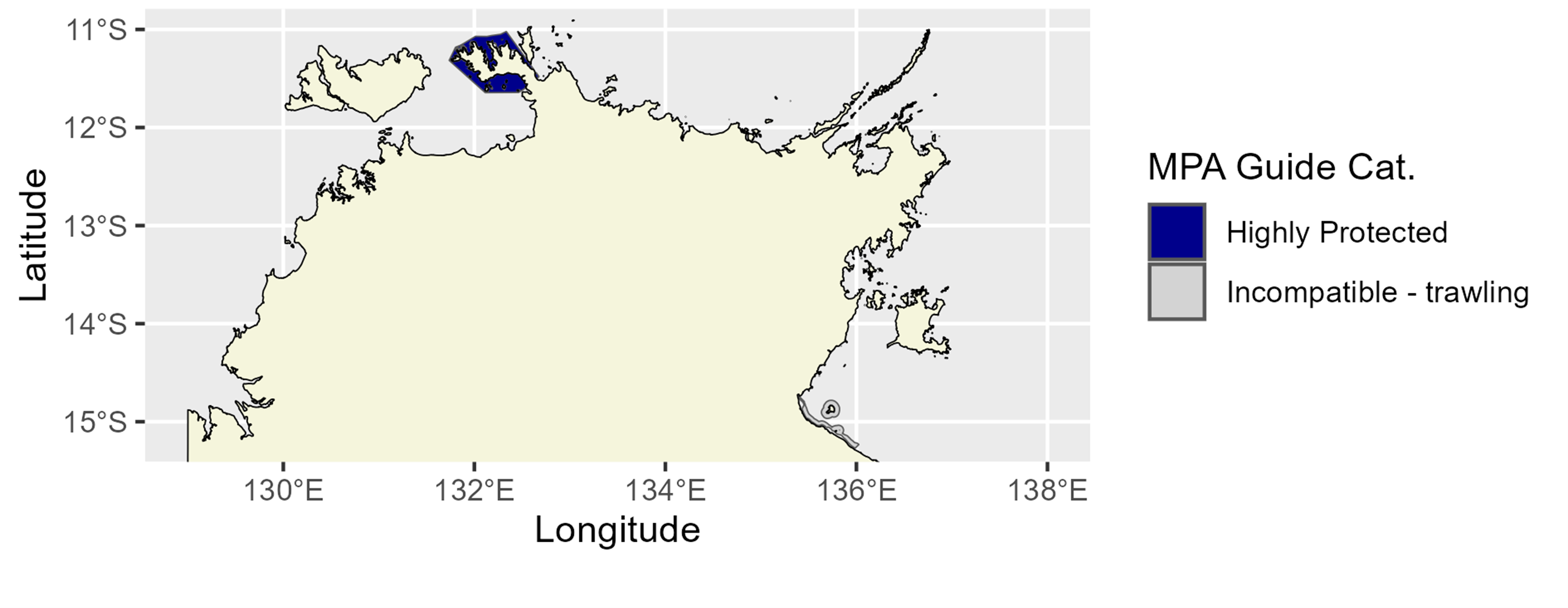

Supplement: S3 Fig — All data relating to MPAs data are sourced from the Collaborative Australian Protected Area Database (CAPAD) dataset for 2022 [37]. Australian jurisdictional boundaries are publicly available and sourced from Geosciences Australia (based on the Geocentric Datum of Australia 2020, GDA2020). Mapping was completed using spatial package sf [38,39] within the R Programming Framework [120]. All data are publicly available, and access to the data is via the Creative Commons Attribution (CC-BY) licence model:–CC By 4.0 International (https://creativecommons.org/licenses/by/4.0/). (TIF) [file pone.0307324.s004.tif]

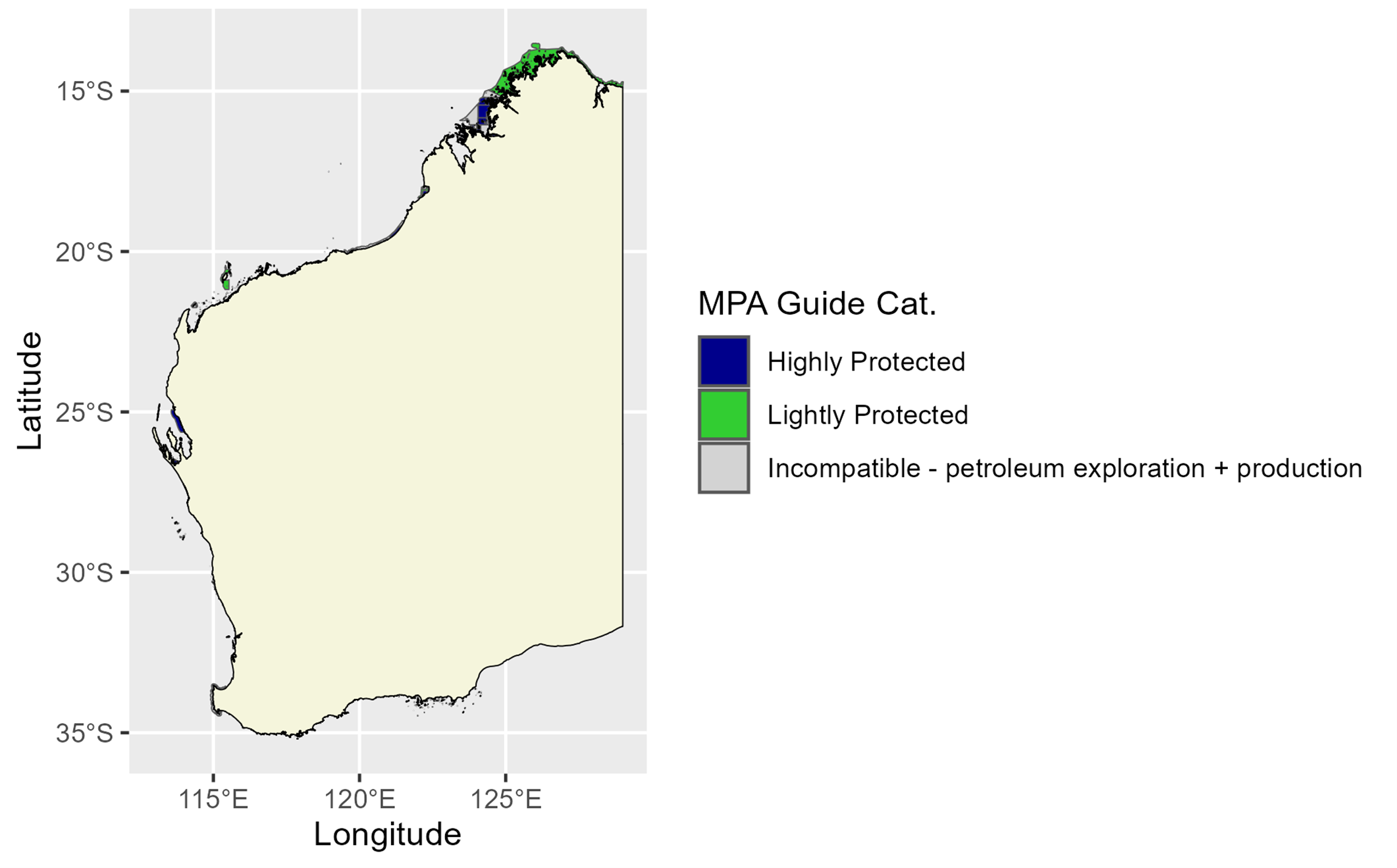

Supplement: S4 Fig — All data relating to MPAs data are sourced from the Collaborative Australian Protected Area Database (CAPAD) dataset for 2022 [37]. Australian jurisdictional boundaries are publicly available and sourced from Geosciences Australia (based on the Geocentric Datum of Australia 2020, GDA2020). Mapping was completed using spatial package sf [38,39] within the R Programming Framework [120]. All data are publicly available, and access to the data is via the Creative Commons Attribution (CC-BY) licence model:–CC By 4.0 International (https://creativecommons.org/licenses/by/4.0/). (TIF) [file pone.0307324.s005.tif]

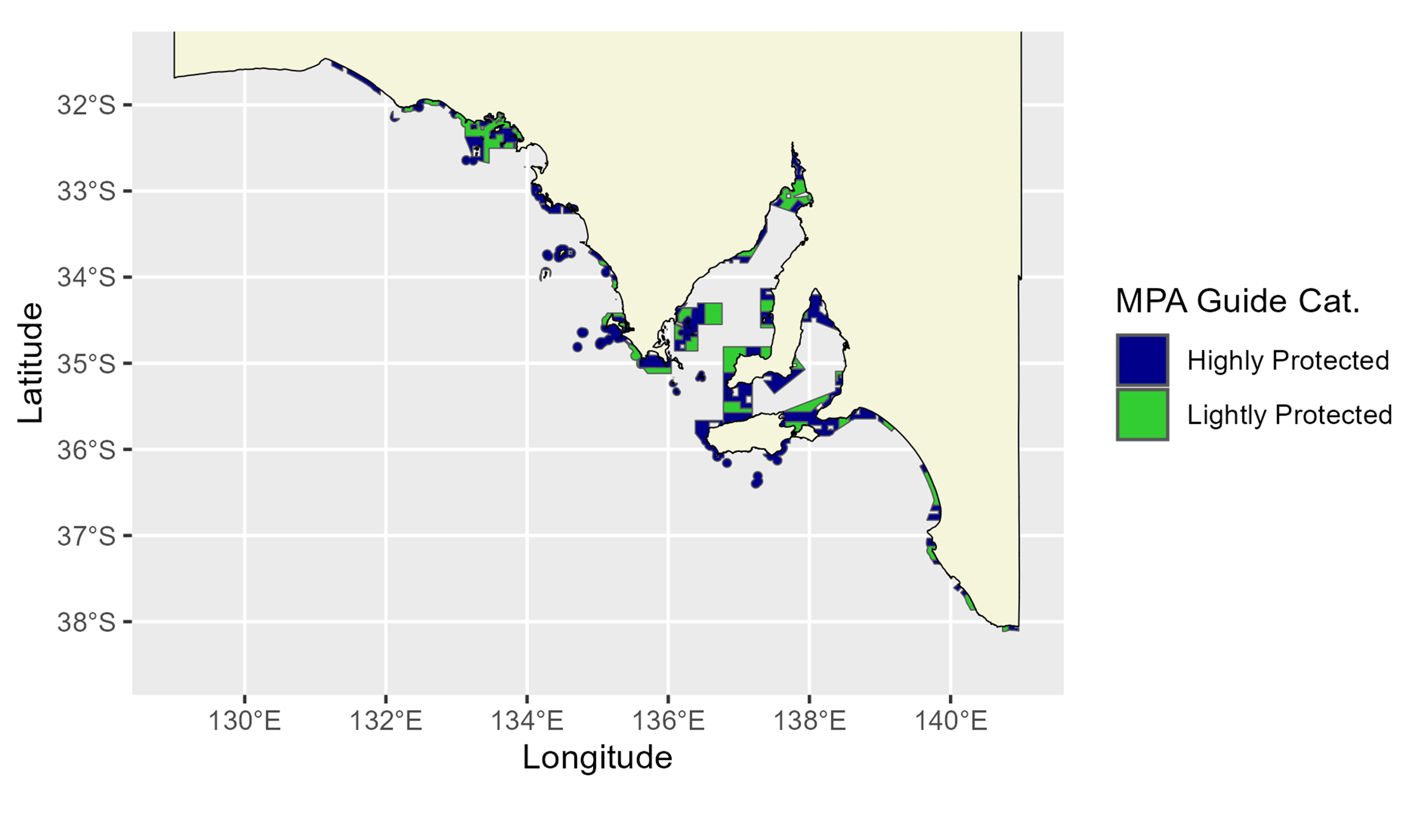

Supplement: S5 Fig — All data relating to MPAs data are sourced from the Collaborative Australian Protected Area Database (CAPAD) dataset for 2022 [37]. Australian jurisdictional boundaries are publicly available and sourced from Geosciences Australia (based on the Geocentric Datum of Australia 2020, GDA2020). Mapping was completed using spatial package sf [38,39] within the R Programming Framework [120]. All data are publicly available, and access to the data is via the Creative Commons Attribution (CC-BY) licence model:–CC By 4.0 International (https://creativecommons.org/licenses/by/4.0/). (TIF) [file pone.0307324.s006.tif]

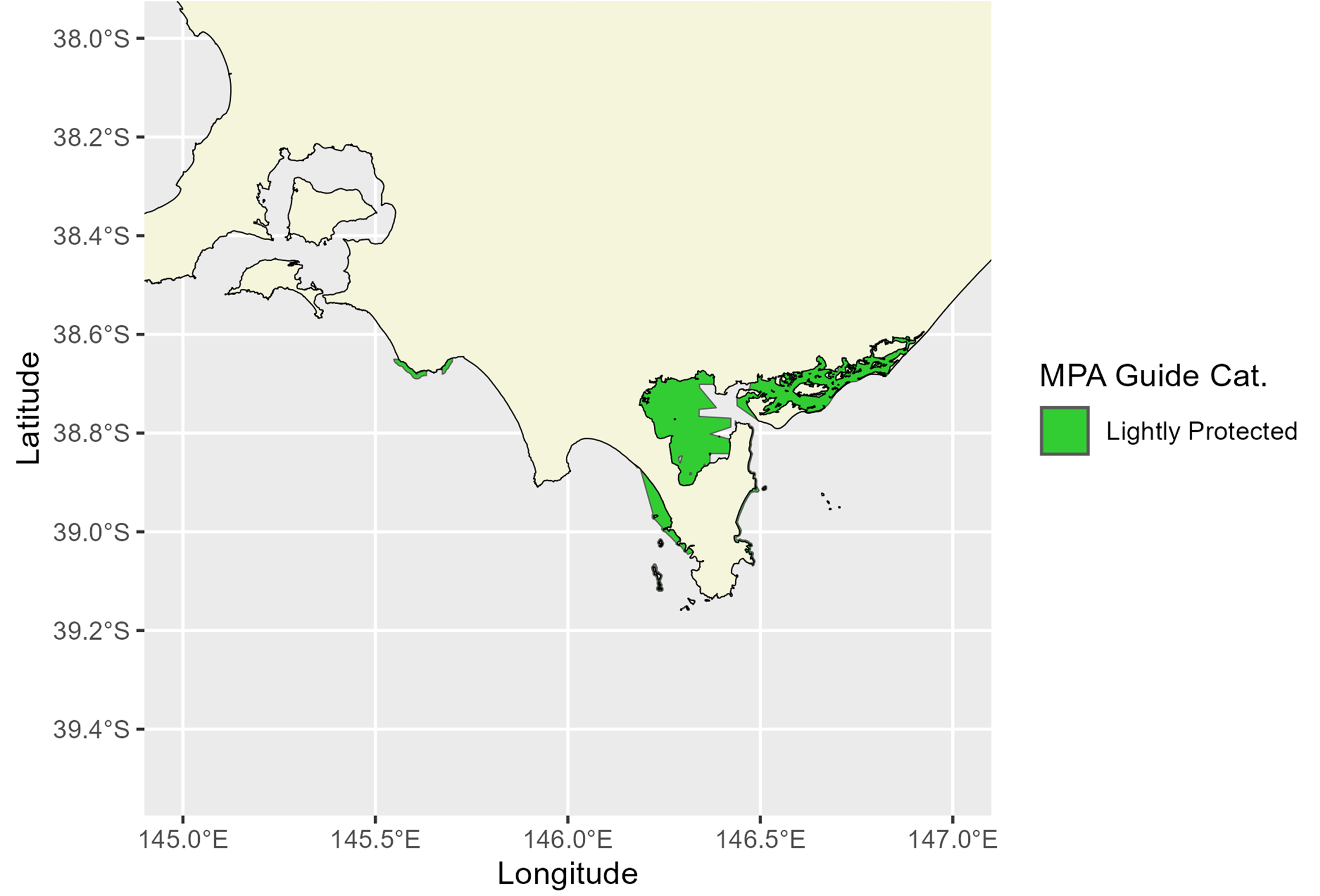

Supplement: S6 Fig — All data relating to MPAs data are sourced from the Collaborative Australian Protected Area Database (CAPAD) dataset for 2022 [37]. Australian jurisdictional boundaries are publicly available and sourced from Geosciences Australia (based on the Geocentric Datum of Australia 2020, GDA2020). Mapping was completed using spatial package sf [38,39] within the R Programming Framework [120]. All data are publicly available, and access to the data is via the Creative Commons Attribution (CC-BY) licence model:–CC By 4.0 International (https://creativecommons.org/licenses/by/4.0/). (TIF) [file pone.0307324.s007.tif]

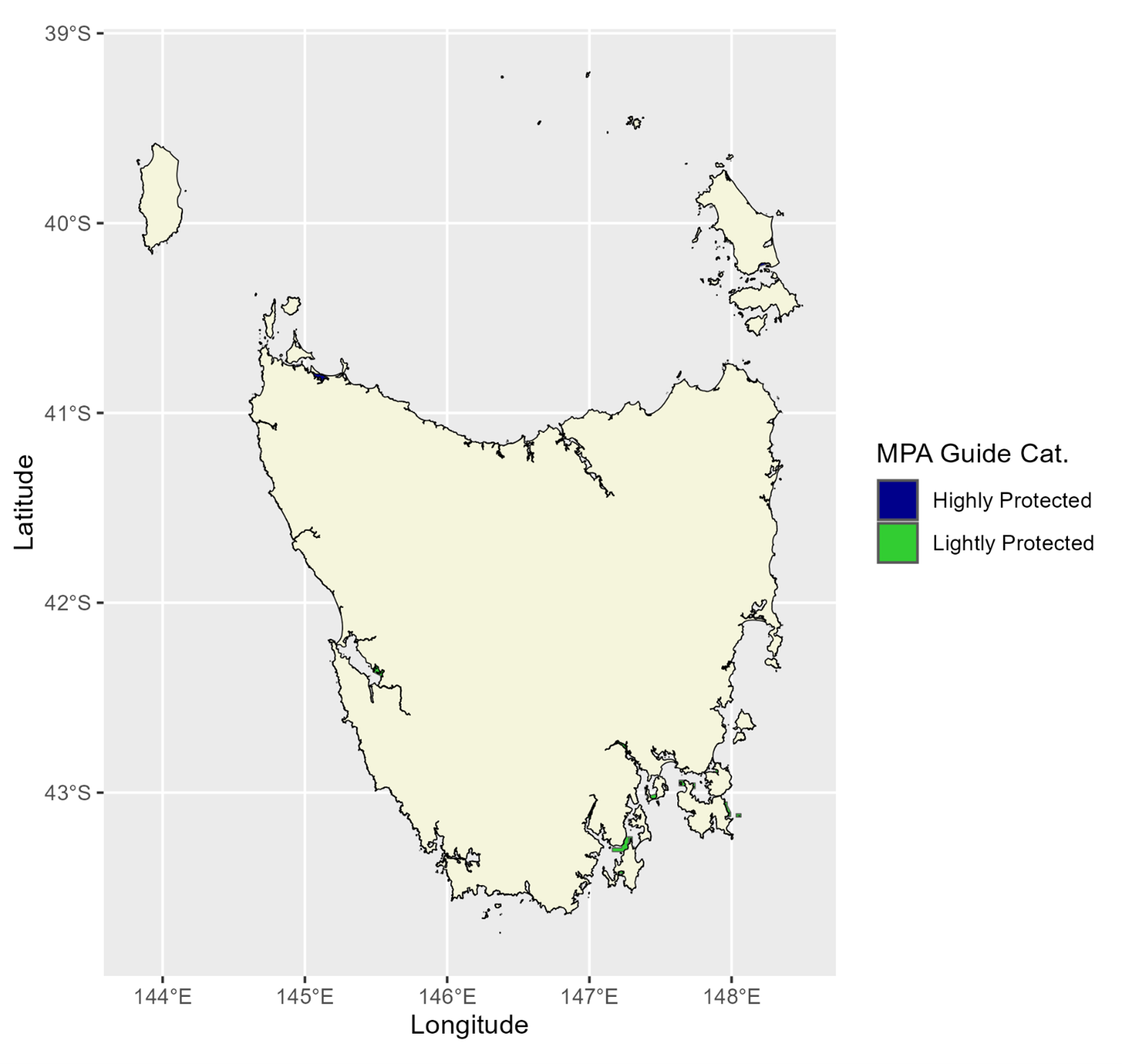

Supplement: S7 Fig — All data relating to MPAs data are sourced from the Collaborative Australian Protected Area Database (CAPAD) dataset for 2022 [37]. Australian jurisdictional boundaries are publicly available and sourced from Geosciences Australia (based on the Geocentric Datum of Australia 2020, GDA2020). Mapping was completed using spatial package sf [38,39] within the R Programming Framework [120]. All data are publicly available, and access to the data is via the Creative Commons Attribution (CC-BY) licence model:–CC By 4.0 International (https://creativecommons.org/licenses/by/4.0/). (TIF) [file pone.0307324.s008.tif]

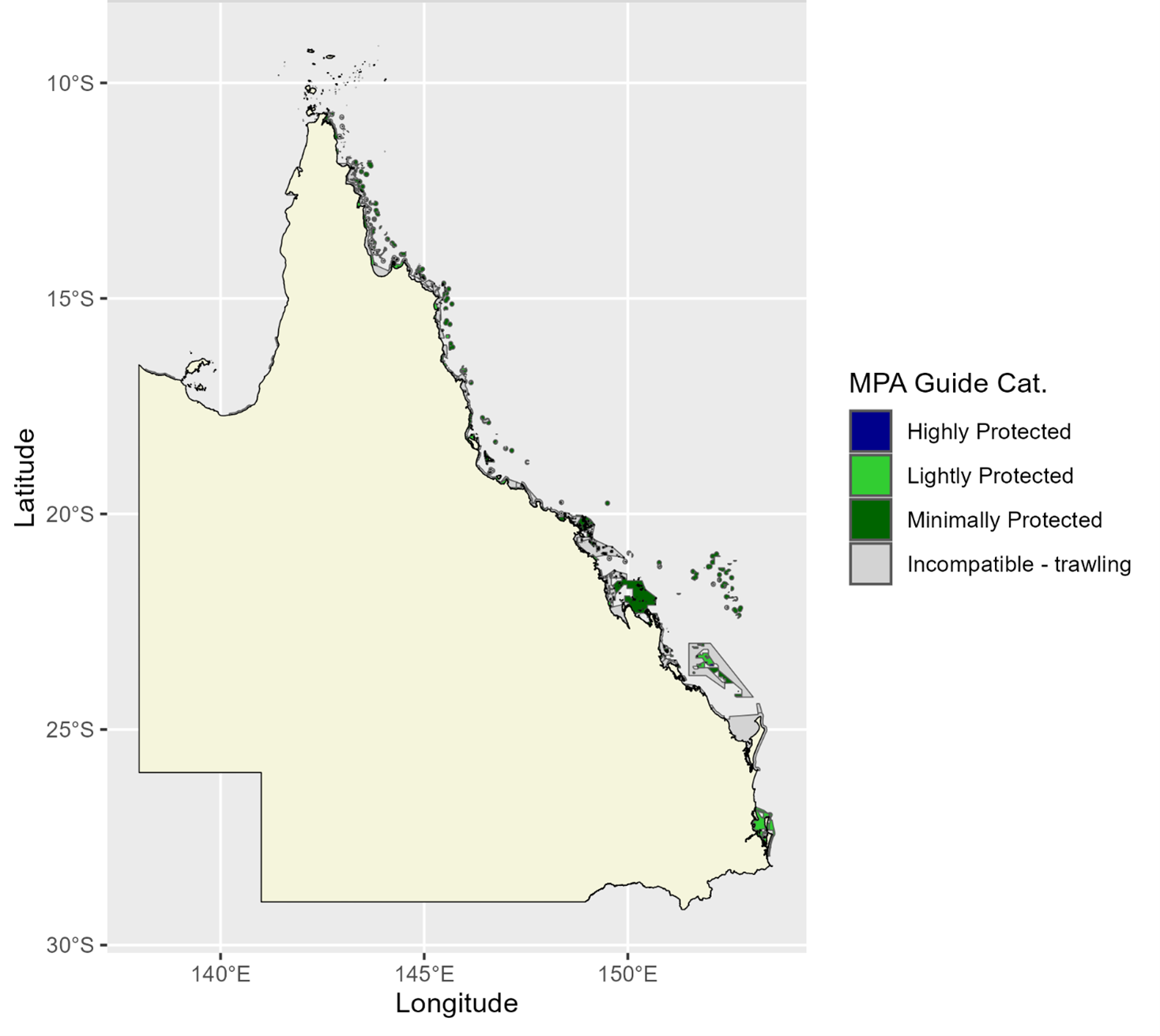

Supplement: S8 Fig — All data relating to MPAs data are sourced from the Collaborative Australian Protected Area Database (CAPAD) dataset for 2022 [37]. Australian jurisdictional boundaries are publicly available and sourced from Geosciences Australia (based on the Geocentric Datum of Australia 2020, GDA2020). Mapping was completed using spatial package sf [38,39] within the R Programming Framework [120]. All data are publicly available, and access to the data is via the Creative Commons Attribution (CC-BY) licence model:–CC By 4.0 International (https://creativecommons.org/licenses/by/4.0/). (TIF) [file pone.0307324.s009.tif]
